# CCI: A Consensus Clustering-Based Imputation Method for Addressing Dropout Events in scRNA-Seq Data

**DOI:** 10.3390/bioengineering12010031

**Published:** 2025-01-03

**Authors:** Wanlin Juan, Kwang Woo Ahn, Yi-Guang Chen, Chien-Wei Lin

**Affiliations:** 1 Division of Biostatistics, Data Science Institute, Medical College of Wisconsin (MCW), Milwaukee, WI 53226, USA; wjuan@mcw.edu (W.J.); kwooahn@mcw.edu (K.W.A.); 2 Department of Pediatrics, Medical College of Wisconsin (MCW), Milwaukee, WI 53226, USA; yichen@mcw.edu

**Keywords:** dropout, imputation, consensus clustering, scRNA-seq

## Abstract

Single-cell RNA sequencing (scRNA-seq) is a cutting-edge technique in molecular biology and genomics, revealing the cellular heterogeneity. However, scRNA-seq data often suffer from dropout events, meaning that certain genes exhibit very low or even zero expression levels due to technical limitations. Existing imputation methods for dropout events lack comprehensive evaluations in downstream analyses and do not demonstrate robustness across various scenarios. In response to this challenge, we propose a consensus clustering-based imputation (CCI) method. CCI performs clustering on each subset of data sampling across genes and summarizes clustering outcomes to define cellular similarities. CCI leverages the information from similar cells and employs the similarities to impute gene expression levels. Our comprehensive evaluations demonstrate that CCI not only reconstructs the original data pattern, but also improves the performance of downstream analyses. CCI outperforms existing methods for data imputation under different scenarios, exhibiting accuracy, robustness, and generalization.

## 1. Introduction

Single-cell sequencing is a revolutionary technique in molecular biology and genomics that focuses on studying individual cells in isolation and reveals the cellular heterogeneity. In traditional bulk RNA sequencing (RNA-seq) data, where RNA is extracted from a population of cells and analyzed together, the measured RNA expression levels represent an average of all the cells in the population [1]. Single-cell RNA sequencing (scRNA-seq) enables the profiling of the RNA contents of each single cell separately and achieves distinct expression profiles for different cell types, providing insights into complex cellular diversity and heterogeneity. However, scRNA-seq data exhibit a higher level of noise than bulk RNA-seq data, mainly due to the issue known as dropouts [2]. Dropouts refer to the phenomenon where observed expression levels are very low or even zero for certain genes due to technical limitations. Thus, scRNA-seq data are often highly sparse and zero-inflated. Dropout events can be viewed as one type of missing value problem. Traditional statistical models for missing data imputation often rely on the assumption of missing at random or missing completely at random. However, this assumption does not hold for dropouts in scRNA-seq data, because genes with lower expression levels tend to experience higher chances of dropout events. Thus, imputation methods for scRNA-seq data should hold the assumption of missing not at random [3].

Dropout events can significantly impact the downstream analyses and the interpretation of scRNA-seq data. Various methods have been developed to deal with dropouts and improve the accuracy of scRNA-seq data. These approaches can be broadly classified into two categories [4]. Methods in the first category aim to borrow and summarize information from neighbors such as similar cells or similar genes. The neighbors are typically defined based on similarity measures such as distance metrics or co-membership in clustering. For example, RESCUE [5] uses a bootstrap sampling method to infer the expression distribution of each gene in each cell from neighboring cells, and imputes by using the distribution mean. scImpute [6] learns dropout probability for each gene in each cell by fitting a mixture model and imputes by borrowing information on the same gene from neighboring cells. Methods in the second category denoise the entire dataset using parametric models. DCA [7] uses a deep-learning-based autoencoder to denoise the scRNA-seq data by using zero-inflated negative binomial likelihood as loss function. MAGIC [8] constructs a Markov affinity matrix based on cell-by-cell distance, and achieves the imputed output based on the exponentiated Markov matrix.

Despite the rich literature, these methods lack evidence of effectiveness under different scenarios. Different methods evaluate the performance by using distinct simulation settings, and it is unclear if the specific setting indeed favors the performance reported. Specifically, the evaluation solely relies on simulation analyses with one specific level of dropouts and differential expression (DE) signals [5,6,9]. As a result, directly comparing the imputation performance across methods is challenging due to the usage of unique settings from each method. The generalization of these methods across diverse scenarios remains unclear. Furthermore, the evaluation for how dropouts affecting downstream analyses is very limited from these methods, as Table 1 shows. While many imputation methods focus on clustering, other critical downstream analyses such as DE analysis are mostly ignored. In addition, most methods only focus on the imputation performance of some specific genes, overlooking the general view of imputation performance.

In response to these challenges, we propose a robust imputation method named Consensus Clustering-based Imputation (CCI). CCI utilizes consensus clustering algorithm [10] to define cell-to-cell similarity, which robustly leverage information from neighboring cells and impute gene expression levels. CCI belongs to the first category mentioned in Table 1. Section 2 describes the overview of the CCI algorithm, and provides simulation analysis using a synthetic dataset and real dataset example. We summarize our findings in Section 4. Section 5 includes mathematical details of the CCI algorithm (in Section 5.1), as well as other technical details.

## 2. Results

### 2.1. Overview of the CCI Method

As introduced in Section 1, the first category of the imputation methods leverages the information of the neighboring cells with similar transcriptome patterns to impute the dropout events. However, defining cell-to-cell similarity can be a challenge, especially given the highly sparse scRNA-seq data. Clustering algorithms are used to define cellular similarity, where this process is also known as cell type identification, as cells that share similar transcriptome patterns are likely to belong to the same cell types.

CCI employs a robust measure of cellular similarity by utilizing a consensus clustering algorithm to impute dropout events. The main idea behind consensus clustering is that, instead of relying on one single run of clustering result, it generates multiple clustering results and then identifies clustering patterns that consistently appear across multiple runs, therefore improving the reliability of the identified clusters by reducing the variability.

**Figure 1:**
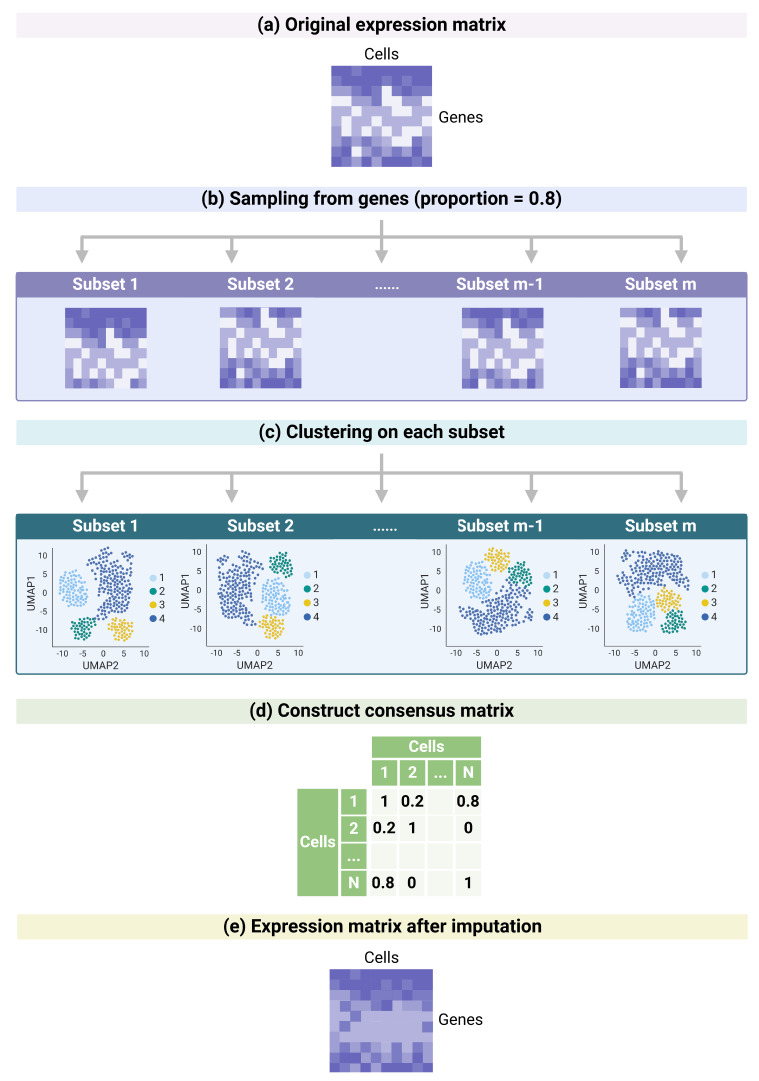
Pipeline of consensus clustering-based imputation (CCI). Created in BioRender. Juan, W. (2025) https://BioRender.com/s37k569 (accessed on 29 December 2024). (**a**) The original expression matrix from scRNA-seq data. The rows represent the genes and the columns represent the cells. Darker color represents higher expression level. (**b**) Sample proportion of genes for *m* times from the original expression matrix to derive *m* subset matrices. The sampling proportion is prespecified. Here, we use 0.8 for illustration. (**c**) Perform clustering algorithm on each subset and derive *m* different consensus matrices accordingly. (**d**) Construct the consensus matrix from *m* co-membership matrices. Each entry represents the frequency that two cells belong to the same cluster. (**e**) The expression matrix after imputation. Zero counts in the original expression matrix are imputed based on the consensus matrix, which reflects cell similarities learned from the consensus clustering results.

The workflow of CCI is illustrated in Figure 1. It begins by subsampling a prespecified proportion of genes to create a subset of the original expression matrix (Figure 1a). Here, we sample 80% of the genes in Figure 1b as an example. This step is repeated multiple times, generating different subsets from the original data. For each subset, a clustering algorithm (such as K-means or shared nearest-neighbor [11], with details of clustering methods in Section 5.8), is applied, and a co-membership matrix is constructed to record relationships between cells, where a value of 1 indicates that two cells belong to the same cluster, and a value of 0 indicates that they do not (Figure 1c). After repeating this process for *m* different subsets, we summarize across *m* different co-membership matrices from *m* clustering outcomes into one consensus matrix (Figure 1d) by summing up *m* co-membership matrices and dividing by *m*. This consensus matrix represents the frequency of any pair of cells identified in the same cluster across the *m* different clustering runs. For example, the entries in the *i*-th row of the consensus matrix can be viewed as the similarity scores between the *i*-th cell and all other cells in the dataset, where a value of 1 refers to the case that two cells are always clustered together and a value of 0 refers to two cells are always in different clusters.

To impute potential dropouts (zero counts) in the expression matrix, CCI leverages these similarity scores. For a given gene, zero counts in the *i*-th cell are imputed by weighted averaging of the expression of neighboring cells (Figure 1e), with the weights determined by the similarity scores from the consensus matrix. This approach allows a data-driven method for imputation, enabling dropout events to be addressed in a biologically meaningful way.

Detailed mathematical details of the CCI algorithm can be found in Section 5.1. The parameters in the algorithm, including sampling proportion and number of samples, are discussed in Section 5.2.

### 2.2. CCI Enhances Clustering Performance

We begin by conducting simulation studies to evaluate the imputation performance of CCI. We generate the synthetic data by using R package *Splatter* [12]. Data without any dropouts are simulated with varying levels of differential expression (DE) signals between cell groups (weak, moderate, and strong) to serve as ground truths. We then generate dropouts at different strengths (weak, moderate, and strong). These combinations of DE signals and dropout levels allow us to generate various data patterns that capture a wide range of realistic scRNA-seq data scenarios. The data after adding dropouts serve as the input for CCI and other competing methods such as RESCUE, scImpute, and DCA. Detailed simulation settings and setup for each competing method can be found in Section 5.3. Details of the implementation of the competing methods are provided in Section 5.4.

In scRNA-seq data analysis, clustering cells into distinct groups is one of the key objectives, as it helps uncover cellular heterogeneity. Dimension reduction techniques such as principal component analysis (PCA), tSNE [13], and UMAP [14] are commonly used to visualize these clustering patterns in low dimensions. To quantify the clustering performance of methods for addressing dropout events, we use two metrics, adjusted Rand index (ARI) and compactness, to assess the clustering performance. ARI measures the agreement between the true clustering labels (underlying truth) and identified clustering labels from shared nearest-neighbor (SNN) [15], while compactness evaluates the relative separation between the identified clusters. The closer the values are to 1, the more favorable the results indicated by both indices. Detailed descriptions of these evaluation methods can be found in Section 5.5.

**Figure 2:**
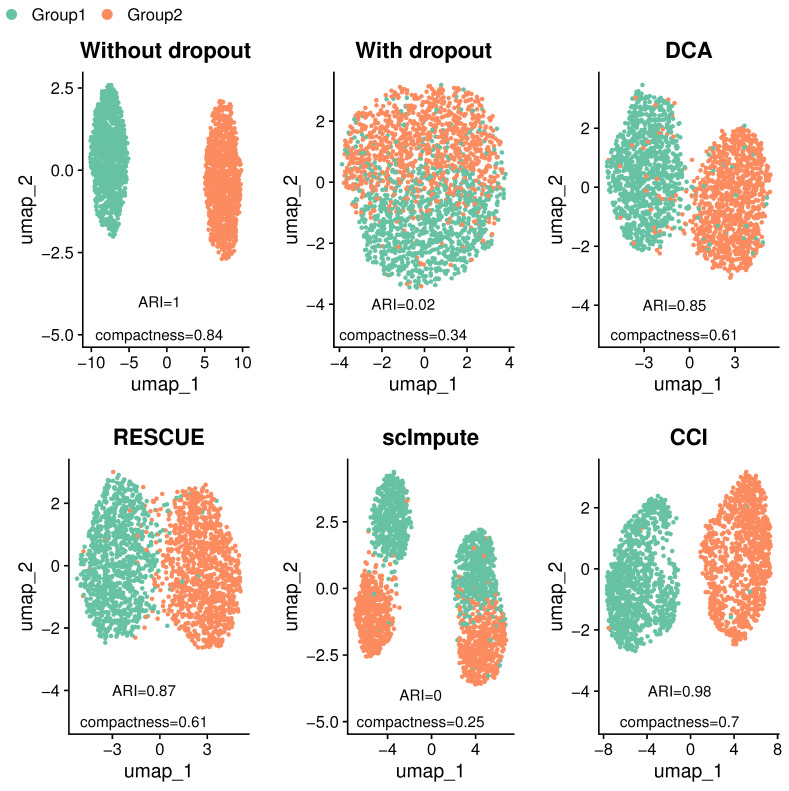
UMAP plots of the simulated data before and after imputation. Synthetic data before imputation include the raw data without dropouts and the one with dropouts. Data with dropouts are used as input for imputation methods, including DCA, RESCUE, scImpute, and CCI. ARI and compactness are calculated based on the clustering outcomes after imputation compared with the underlying clustering labels. Data without dropouts show distinctive difference in two groups, while data with dropouts (before imputation) diminish the underlying clustering pattern. Among all methods, CCI demonstrates the best recovery of the underlying clustering pattern and outperforms other methods, with the highest ARI and compactness.

To demonstrate how CCI works, we present one specific example under a simulation setting with weak DE signals and moderate dropout levels. The definitions of these signal and dropout levels are provided in Section 5.3 and can be visualized in Figure 2, where UMAP plots are used to show clustering results, along with ARI and compactness displayed. Simulated data without dropouts exhibit a well-separated clustering pattern, with high compactness (0.84) and perfect ARI (1). However, when dropouts are introduced, the two-group clustering structure becomes less distinct with significantly lower ARI (0.02) and compactness (0.34). The data with dropouts are then used as input for all imputation methods. DCA and RESCUE are able to partially recover the clustering structure, but small portions of cells remain misclassified or overlap between groups, with ARI as 0.85 and 0.87, respectively. scImpute fails to separate the two groups and, thus, performs poorly in this example, with an ARI close to 0, suggesting that the clustering results are nearly random. In contrast, CCI recovers the underlying clustering pattern (data without dropouts) well with an ARI of 0.98. CCI not only clearly separates the two cell groups, but also pushes the two groups in the opposite direction, resulting in minimal overlap (compactness 0.7).

Among all the methods tested, CCI achieves the highest ARI and compactness, reflecting its superior ability to restore the underlying clustering pattern even under a challenging condition with weak DE signals and moderate dropout levels.

### 2.3. CCI Improves DE Analysis

Differential expression (DE) analysis is an important downstream analysis to identify cluster marker genes that are uniquely expressed in a specific cell population/cluster. However, many existing imputation methods often overlook DE analysis, as Table 1 summarizes. Here, DE analysis is performed based on the data after imputation by CCI and other competing methods using *FindMarkers* function in R package *Seurat* after the clustering analysis. By default, this function performs the Wilcoxon rank-sum test to identify DE genes between two identified clusters of cells, noting that clusters are determined after imputation from SNN. The declared DE genes (adjusted *p*-value <0.05, log fold-change of the average expression ≥0.2) are then compared with the ground truth of DE genes from simulation settings as a benchmark. We quantify the performance using numerical measures such as Jaccard index, sensitivity, and specificity. Jaccard index measures the overlap between the true and declared DE genes, with a higher value close to 1 indicating greater similarity between the two. Sensitivity and specificity are also calculated to assess the methods’ ability to detect true DE genes and avoid false positives. Details for these metrics are provided in Section 5.5.

**Figure 3:**
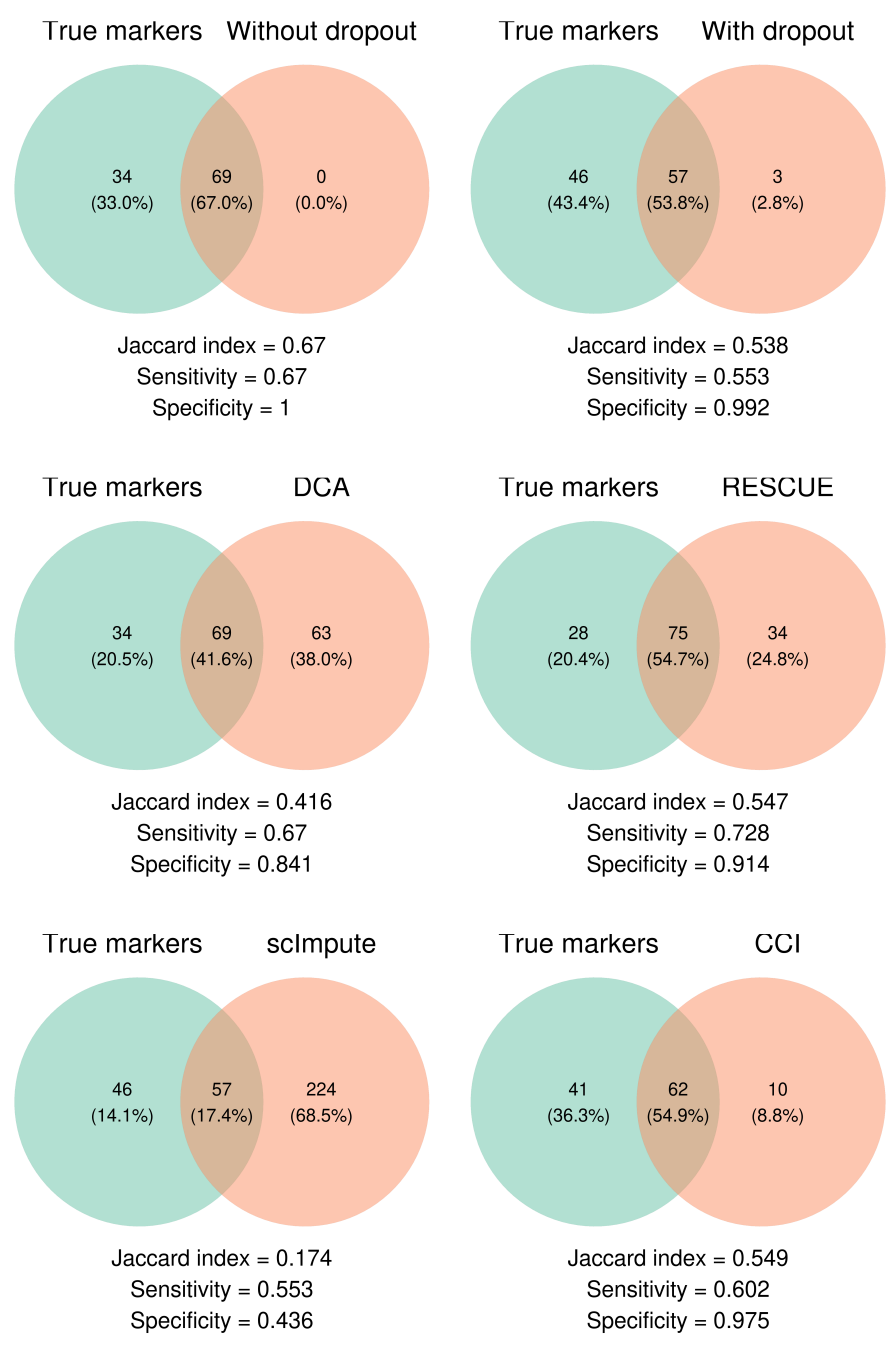
Venn diagrams to evaluate DE analysis under weak signal and moderate dropout setting. Jaccard index, sensitivity, and specificity are calculated to compare between the true DE genes and declared DE genes after clustering analysis. CCI outperforms the other imputation methods regarding Jaccard index and specificity, while keeping comparable performance of sensitivity.

We continue to demonstrate the performance of CCI using the example under weak DE signals and moderate dropout levels. As shown in Figure 3, it displays the Venn diagrams that compare between the true and declared DE genes, along with the calculated Jaccard index, sensitivity, and specificity for each method. There are 103 true DE genes simulated in this particular example. It is worth mentioning that even the data without dropouts fail to capture all true DE genes, which only declares 69 out of 103 DE genes, indicating that the DE signals are, indeed, weak as we design. After introducing dropouts to the data and applying imputation methods, all methods experience a notable decline in performance metrics. DCA declares 69 true DE genes but also 63 false DE genes, resulting in a relatively low specificity, while scImpute declares the most false positives. Both CCI and RESCUE show improvements in the Jaccard index compared to the data with dropouts (before imputation), demonstrating the improvement in DE analysis. Note that CCI stands out by declaring only 10 false DE genes (highest specificity across all imputation methods) while keeping a similar level of sensitivity, which can further help reduce the noise and produce better downstream analysis, such as pathway analysis, to reveal true biological context.

### 2.4. CCI Recovers the Gene Expression Pattern from Dropouts

As the goal of imputation or denoising is to reflect the underlying data structure and biological context, in addition to revealing cell clusters, we also expect the imputed or denoised data to reveal the underlying gene expression pattern. In particular, marker genes, which are important for distinguishing different cell types, should demonstrate expression levels after imputation approximate to the underlying truth. To evaluate this aspect in different imputation methods for each gene, we perform correlation analysis to quantify the similarity between the imputed gene expression data and the underlying ground truth. Given the high sparsity and skewness of scRNA-seq data, Spearman’s correlation, as a rank-based nonparametric measurement for assessing the monotonic relationship, is preferred over Pearson correlation, which is prone to being affected by outliers. A higher correlation value close to 1 refers to the successful recovery of a true gene expression pattern.

Using the same example under weak DE signals and moderate dropouts level, we calculate the average of Spearman’s correlation across true DE genes between the underlying truth data without dropouts compared to the imputed or denoised data. After adding dropouts, the average Spearman’s correlation drops to only 0.310, showing the impact due to dropouts that disrupt the gene expression pattern. We apply CCI and other competing methods to the data with dropouts and compare the Spearman’s correlations. CCI achieves the highest average Spearman’s correlation 0.380 across all DE genes, surpassing the correlation between data with dropouts and underlying truth, demonstrating the improvement in correlation analysis. However, the imputation methods RESCUE and scImpute only achieve average Spearman’s correlations of 0.286 and 0.274, respectively. DCA, as a denoising method, alters not only the dropouts but also the nondropout values, resulting in a lower correlation of 0.268 in this setting.

**Figure 4:**
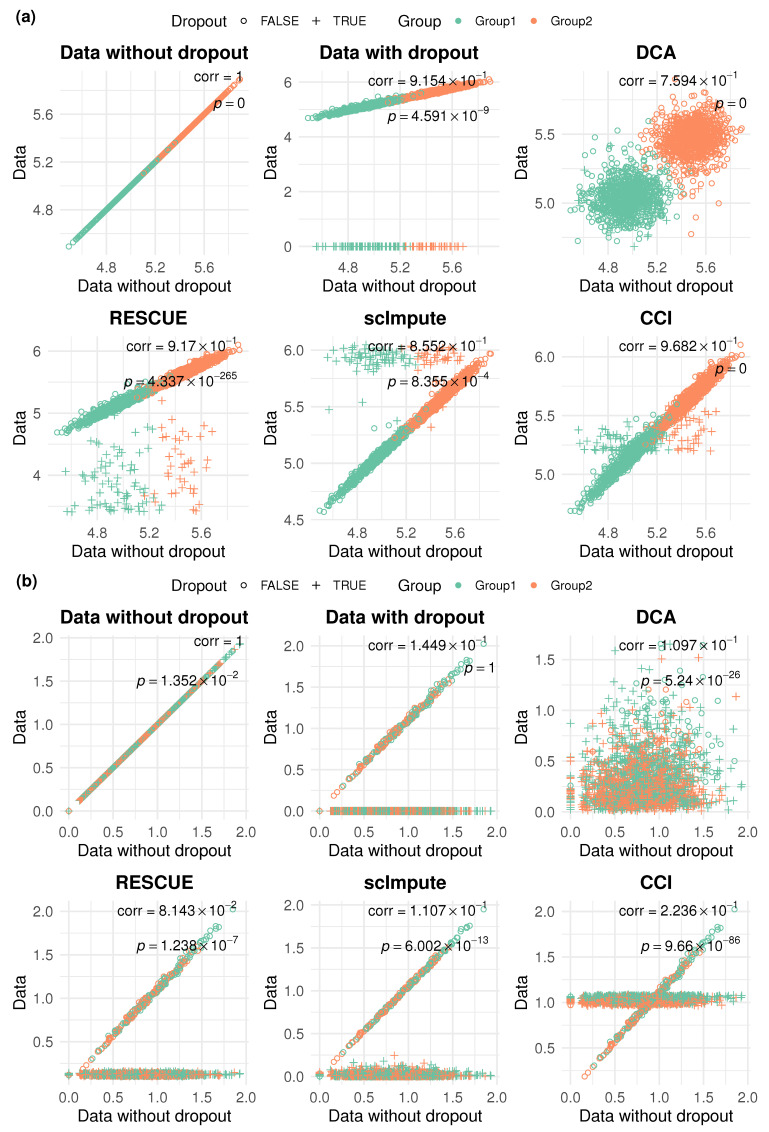
Scatter plots for marker genes to evaluate imputation performance at the single-gene level under weak signal and moderate dropout setting. Data on the x-axis show the data of ground truth without dropouts, and data on the y-axis show the data with dropouts before and after imputation. Two colors represent the true group labels. Dots represent nondropout values, while crosses represent dropouts. (**a**) An example with a “strong” marker, where CCI recovers the data perfectly with a *p*-value close to 0 and Spearman’s correlation close to 1. (**b**) An example with a “weak” marker, where CCI effectively raises the dropouts around the mean expression and restores the group differences in expression.

In Figure 4, we show scatter plots for two marker genes as examples. The two distinct colors represent two simulated groups of data, with crosses indicating cells with dropouts and dots representing cells without dropouts. A good imputation method should produce scatter plots that closely align with the 45-degree line, meaning high similarity to the underlying gene expression; in other words, recovering well from dropouts. In addition, we also calculate *p*-values using the Wilcoxon rank-sum test to assess the difference in expression levels between the identified groups from clustering analysis, while Spearman’s correlation is computed to show the similarity for the specific marker gene.

In Figure 4a, we use a “strong” marker (top sixth variable gene) as an example. The *p*-values for data without or with dropout are both less than 0.05, indicating that two groups show significant differences in expression levels even after introducing a moderate level of dropouts. Furthermore, data with dropouts demonstrate high similarity to the underlying true gene expression level, with a high Spearman’s correlation (0.92). All imputation methods achieve Spearman’s correlations higher than 0.75 and *p*-value less than 0.05, but the scatter plots show very different patterns. DCA denoises the entire expression matrix, leading to a relatively low correlation, as we mentioned before. RESCUE raises the dropout values but is still significantly lower than the mean expression level. On the contrary, scImpute lifts the dropouts significantly higher than the mean expression level. In this example, CCI produces the highest correlation level (0.97) and the pattern is closest to the 45-degree line, highlighting the great similarities between the imputed data and the underlying true gene expression level.

In Figure 4b, we use a “weak” marker (top 92nd variable gene) as another example. Only data without dropouts show significant difference between two clusters (*p*-value = 0.01); data with dropouts have a *p*-value close to 1 and Spearman’s correlation drops to only 0.145, indicating that the difference for two groups is diminished due to dropouts. Competing methods partially recover the group differences with *p*-values less than 0.05, while the Spearman’s correlation is lower than 0.2. CCI shows a significant *p*-value and achieves the highest Spearman’s correlation (0.24), demonstrating the ability to recover the original data even for this “weak” marker.

### 2.5. CCI Exhibits Robustness Under Various Scenarios

**Figure 5:**
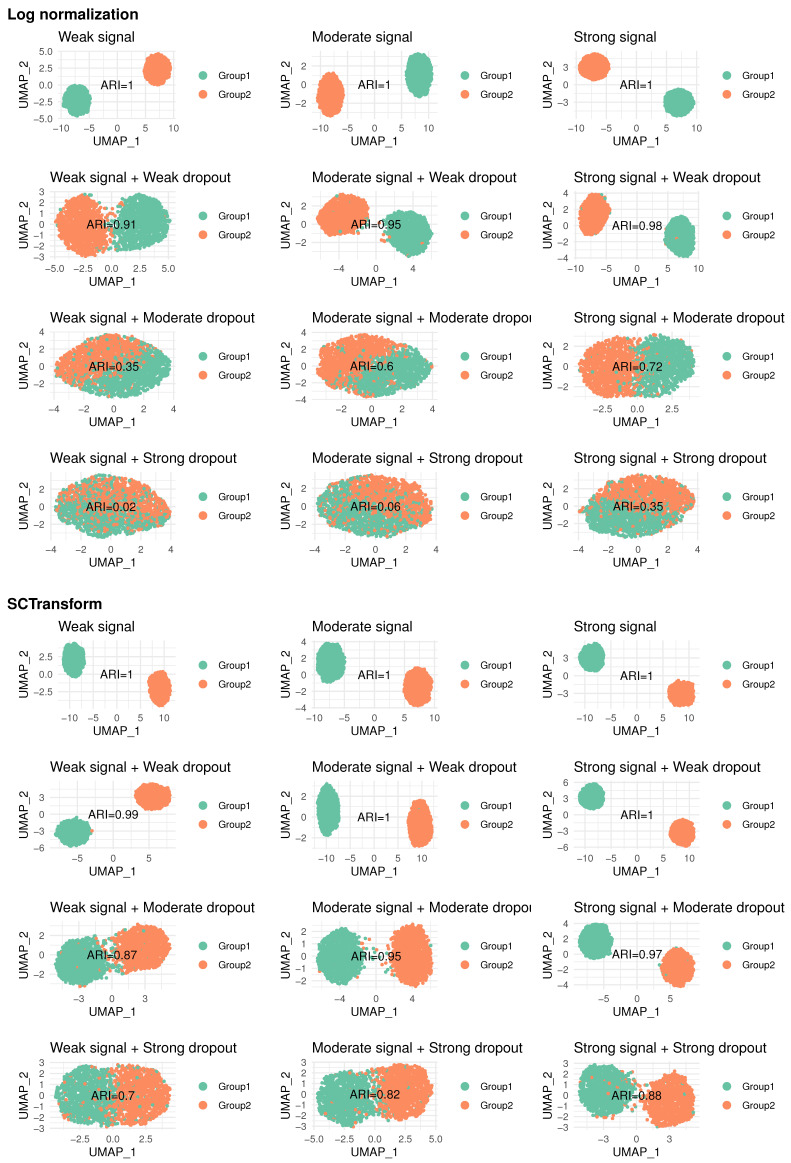
UMAP plots of nine simulation settings for different levels of signal and dropout under both SCTransform and log normalization. ARI is displayed in the center of each plot to show the similarity between the data and the ground truth.

In the above examples, we use example data generated under weak DE signal and moderate dropout to show how CCI enhances downstream analyses including clustering, DE analysis, and single-gene evaluation. As mentioned in Section 1, a robust method for addressing dropouts should perform well under various scenarios rather than one specific setting. Here, we define three levels of DE signals (as weak, moderate, and strong) and three levels of dropouts (as weak, moderate, and strong). These combinations result in nine distinct simulation settings in total, each representing a different pattern in the data, as shown in Figure 5. More details of simulation settings are provided in Section 5.3.

For each of the nine simulation settings, we generate 200 different datasets and evaluate the performance of CCI and competing methods. Key metrics, including ARI, compactness, Jaccard index, and averaged Spearman’s correlation across true DE genes, are calculated to assess the performance of clustering, DE analysis, and single-gene evaluation, respectively. For each method, we compute the mean and standard deviation of these metrics in 200 repeats. Then, we average the means and standard deviations in nine simulation settings, and summarize these results in Table 2.

Surprisingly, DCA and scImpute perform poorly in clustering, with ARI even lower than the data with dropouts, which are the data before the imputation method is applied. KNN struggles to capture the structure of DE genes, resulting in poor performance in DE analysis, as reflected in the Jaccard index. DCA, by denosing the entire expression matrix, achieves the lowest Spearman’s correlation, indicating that it fails to recover the underlying data structure. RESCUE enhances the clustering analysis with a higher ARI and compactness compared to the data with dropouts; however, it shows little improvement in DE analysis and single-gene evaluation.

In addition, these methods have different performances under different settings. For example, DCA and scImpute achieve a comparable performance in clustering with other methods under weak dropout level but perform much worse than others under moderate or strong dropouts. The results for each of the nine simulation settings are presented separately in the Supplementary Materials (Supplementary Figures S1–S4).

In contrast to competing methods, CCI consistently outperforms the data with dropouts in all three downstream analyses. CCI achieves the highest mean values of all key metrics while maintaining the lowest variation from 200 repeats. Moreover, CCI shows better, or at least comparable, results compared to competing methods under all the nine settings, showing no preference for the simulation settings. These findings highlight the robustness and stability of CCI under different simulation settings and demonstrate its ability to effectively address dropouts in various scenarios.

### 2.6. CCI Adapts to Different Normalization Methods

scRNA-seq data often exhibit systematic variations in sequencing depth across libraries due to technical factors such as inconsistent preparation or limited starting material [16]. Normalization is essential to remove these technical variations and ensure that differences in expression profiles reflect biological factors rather than technical variations. Since normalization influences the downstream analysis, it is important to evaluate how well imputation methods perform under different normalization strategies.

In the above simulation sections, we use the well-adopted log normalization method for both simulated and imputed data. To further assess the robustness of CCI under different normalization methods, we use another well-known normalization method, SCTransform, available in the R *Seurat* package, and evaluate performance across different imputation methods. More investigations about log normalization and SCTransform can be found in Section 5.6.

As Table 3 shows, there are several noticeable differences in clustering results under SCTransform. Specifically, the data with dropouts achieve higher ARI and compactness under SCTransform, as shown in Figure 5. KNN fails completely in clustering performance under SCTransform (ARI = 0.01), while RESCUE performs worse under SCTransform (ARI = 0.676) compared to log normalization (ARI = 0.868). DCA and scImpute perform better under SCTransform, but show no improvement in clustering performance compared to the data with dropouts (data before imputation). In addition, DCA, RESCUE, and scImpute are pretty sensitive to the simulation settings. They achieve ARI greater than 0.8 under weak or moderate dropouts, but perform much worse under strong dropouts (ARI close to 0 under weak or moderate DE signals). CCI, however, continues to deliver the best or comparable performance across methods under nine simulation settings, as reflected in ARI and compactness.

In terms of DE analysis, overall, the Jaccard index drops significantly in Table 3 compared to Table 2 across each method, but CCI still achieves the highest Jaccard index among imputation methods. The performance of Spearman’s correlation in each method remains relatively stable, as shown in Table 2, and, once again, CCI shows the highest Spearman’s correlation.

In summary, CCI still outperforms the competing methods in terms of ARI, compactness, Jaccard index, and Spearman’s correlation, demonstrating its robustness across nine simulation settings under SCTransform normalization. Moreover, as shown in Table 2 and Table 3, the competing methods show substantial differences in performance, indicating that other methods are sensitive to the choice of normalization method. In contrast, CCI exhibits adaptability to both the log normalization and SCTransform methods, maintaining robust performance even though normalization methods have a considerable impact on downstream analyses. The results for each of the nine simulation settings are presented separately in the Supplementary Materials (Supplementary Figures S5–S8).

### 2.7. CCI Improves Downstream Analyses in a Real Dataset

To evaluate the performance of CCI and other competing methods in real dataset, we download a Type-I diabetes mouse dataset that investigates the effect of CD137 (Tnfrsf9) deficiency on regulatory T and CD8 T cells from Gene Expression Omnibus (GEO), accession number GSE269611. Preprocessing and quality control are performed before the imputation analysis on this dataset. Detailed descriptions of the dataset are provided in Section 5.7.

This dataset is highly sparse, where 86% of the original counts are zeros. Since we cannot determine whether the zero counts are real dropout events or not, the underlying ground truths without dropouts remain unknown. Hence, we introduce extra dropouts to the data and use them as input for all methods. After applying imputation methods, we can then evaluate how well the data with extra dropouts can be recovered to the original data without extra dropouts.

Similar to the simulation studies, we introduce three different levels of extra dropouts added to the original real data: (1) no extra dropouts (apply methods directly to the original data), (2) with extra 3.8% dropouts, and (3) with extra 4.9% dropouts. For each dropout level, we simulate 100 independent and identically distributed (i.i.d.) dropout patterns from R Splatter package and add to the original data to serve as input for the imputation methods. These repeated simulations provide a robust framework to assess the accuracy and stability of CCI and other competing methods.

**Figure 6:**
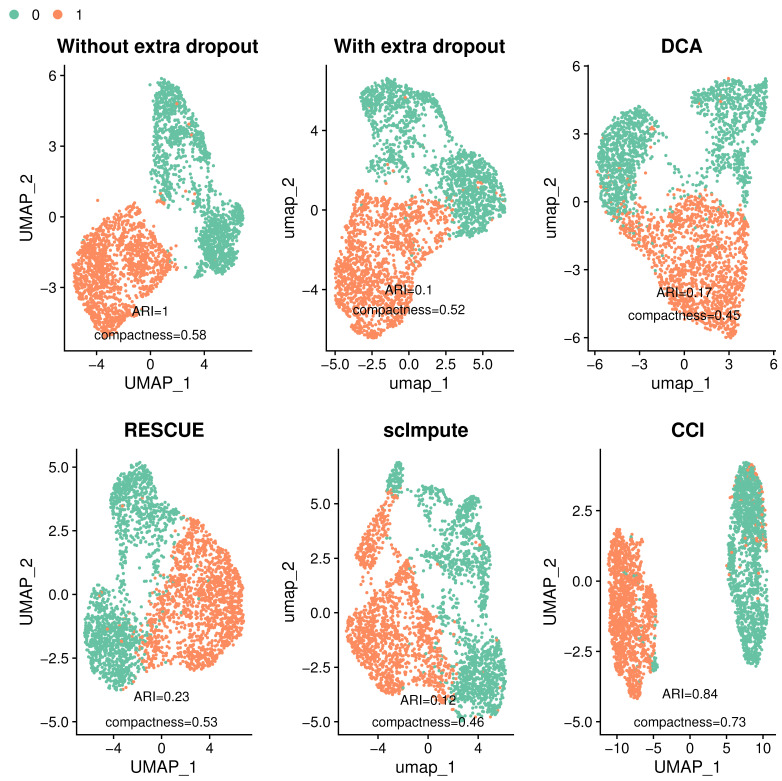
UMAP plots of the data with and without imputation or denoising to show the clustering patterns using a type-1 diabetes mouse dataset. ARI and compactness are calculated based on the predicted clustering outcomes compared with the ground truth clustering labels. Data without dropout show clear separation, while data with dropout significantly reduce the clustering pattern. The competing methods struggle with extra dropouts, while CCI restores the clustering pattern and outperforms other methods, with the highest ARI and compactness.

Figure 6 illustrates one example under an extra 3.8% of dropouts. The original data without extra dropouts display a clear separation, while the clustering pattern becomes obscured after introducing extra dropouts. Competing methods struggle with the extra dropouts and show no separation between groups. CCI, however, demonstrates the ability to recover the underlying clustering pattern. Despite a few misclassified cells, CCI pushes the two groups further apart, resulting in the best ARI and compactness among all methods.

**Figure 7:**
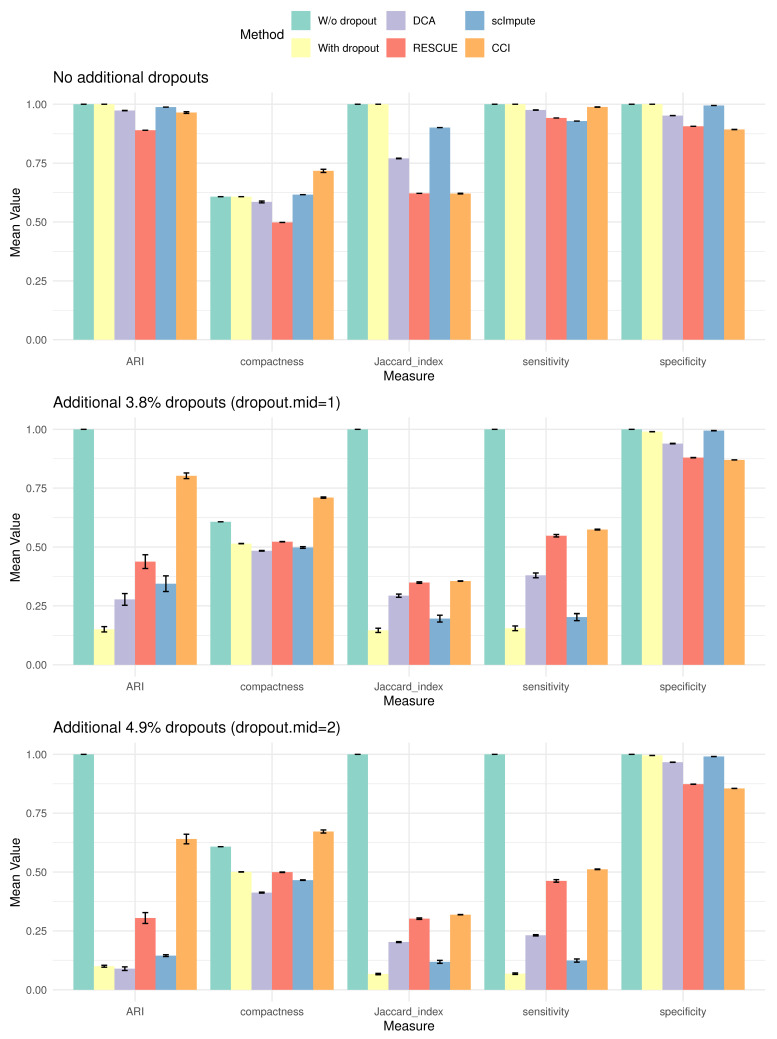
Barplots of key metrics using type-1 diabetes mouse dataset under three dropout scenarios: no additional dropouts, additional 3.8% dropouts, and additional 4.9% dropouts. Mean and standard error are calculated using 100 repeats. While competing methods show degraded performance with increasing dropouts, CCI demonstrates both effectiveness and robustness, maintaining strong performance even under challenging conditions.

We further evaluate clustering and DE analysis under three different levels of dropouts, focusing on key metrics including ARI, compactness for clustering, and Jaccard index, sensitivity, and specificity for DE analysis. Since the true DE genes are unknown, Jaccard index, sensitivity, and specificity are calculated by comparing the declared DE genes of imputed data to those from the original data without extra dropouts. Figure 7 presents barplots of these metrics for method comparison.

When applied directly to the original data without extra dropouts, CCI and competing methods yield comparable results. However, with increasing extra dropout levels, the performances of competing methods decline significantly. In contrast, CCI outperforms other methods in ARI, compactness, Jaccard index, and sensitivity, demonstrating its robustness in imputing dropout events even in challenging scenarios in the real data.

## 3. Conclusions

Although scRNA-seq data analysis provides insights into heterogeneous cell populations, dropout events can significantly corrupt the data, having a substantial impact on downstream analyses. To address dropout events, we propose CCI, a consensus clustering-based imputation method to impute the zero counts in scRNA-seq data.

CCI defines robust neighbors by calculating cellular similarity from different clustering outcomes obtained through multiple subsamplings. CCI then imputes potential dropout events by borrowing information from neighboring cells. Drawing inspiration from ensemble methods like random forest, which combines predictions from multiple weak learners to create a stronger model, CCI aggregates multiple clustering outcomes from random subsets of genes to generate a robust consensus matrix. This ensemble-based framework provides a more reliable clustering result as a foundation for imputation in CCI.

In simulation analysis, we showed that in three types of downstream analyses, including clustering, DE analysis, and gene expression correlation analysis, at the single-gene level, CCI not only outperforms competing methods but also demonstrates consistent performance under different normalization methods such as the log normalization method and the SCTransform method in the preprocessing step. CCI also shows robustness under combinations of different levels of DE signals and dropouts. In NOD mice real data analysis, CCI surpasses competing methods by effectively removing the effect from extra dropouts and recovers the original cell clustering result, demonstrating CCI’s effectiveness in using real datasets with a high number of genes.

Moreover, although CCI performs better under the log normalization method in simulation studies, it also outperforms the competing methods under SCTransform in simulation or real data analysis. Interestingly, under SCTransform, compared to results without imputation (“with dropout”), there seems to be limited improvement after imputation from CCI in our simulation study. However, we show that in real data analysis, CCI outperforms other methods under SCTransform, including results from “with dropout”. These findings demonstrate CCI’s effectiveness under both normalization methods.

As a useful tool for addressing dropout events in scRNA-seq data, CCI offers significant advantages in accuracy, flexibility, and robustness. CCI improves downstream analyses and enables more accurate biological interpretations. This highlights CCI’s potential to significantly advance the understanding of cellular heterogeneity and the underlying biological processes.

## 4. Discussion

Since dropout events significantly corrupt the data and affect the downstream analyses, it is crucial for imputation methods to impute the dropouts while keeping the patterns for nondropout values. While CCI is not a probabilistic model designed to differentiate between the biological zeros with dropouts prior to imputation, its methodology inherently addresses this concern by imputing the zero counts based on cell-to-cell similarities. For example, if a specific gene is lowly expressed or has many zeros across similar cells, it is likely to represent biological zeros. In such cases, CCI imputes these values as very low values or even zeros by employing a weighted average from similar cells that carry essentially mostly zeros, thereby minimizing the risk of incorrectly imputing biological zeros as nonzero values.

CCI is made a flexible framework by providing it with several parameters and options. It allows the specification of gene sampling proportion, gene sampling range, cutoff for defining neighbors, and number of groups for clustering, which balance between local neighborhood and global structure. As for recommendations, a smaller number of groups like two or three is more appropriate for capturing global patterns, which is useful when users aim to capture broad clustering trends or when the aim is to identify major cell populations. A larger number of groups like five or six would prefer to capture local structures, which is beneficial for datasets with more heterogeneous subpopulations. In addition, to choose the genes sampling range in high-dimensional settings, informative genes can be selected by their variability across cells (e.g., using methods like FindVariableFeatures function in R package Seurat) or by prioritizing known biologically relevant markers. In addition, the subsampling scheme can be generalized by not only subsampling in genes but also in cells, which can lead to different performances in key metrics and computational load.

While this paper focuses on the application of CCI to scRNA-seq data, future research could explore its adaptability to other high-dimensional, sparse biological datasets, such as single-cell ATAC-seq or protein expression data, where dropout events also pose analytical challenges. Extending CCI to these modalities could open new avenues for comprehensive multi-omics integration and deepen insights into cellular states across different data types.

## 5. Methods

### 5.1. Details of CCI

The CCI algorithm takes a normalized data matrix as input and follows these steps:

1. A prespecified proportion *p* of genes is sampled and, thus, a subset from the expression matrix is obtained each time. In Section 2, we use proportion p=0.8.
2. Clustering is performed on the subset and a consensus matrix is constructed, where entries are either 0 or 1, denoting if the two cells belong to the same cluster. Here, we use K-means for clustering on the subsets in Step 1 for computational efficiency. Shared nearest-neighbor (SNN) can also be specified as a choice of clustering method. Further details on clustering methods are provided in Section 5.8.
  The (i,j)−th entry of the consensus matrix (CM) is defined as
  (1) $$CM_{ij}=\left\{\begin{array}{cc} 1, & \mathrm{if}\;\mathrm{cell}\;i\;\mathrm{and}\;\mathrm{cell}\;j\;\mathrm{belong}\;\mathrm{to}\;\mathrm{the}\;\mathrm{same}\;\mathrm{cluster};\\ 0, & \mathrm{otherwise}. \end{array}\right.$$
3. Steps 1 and 2 are repeated *m* times. The consensus matrices from each iteration are summed, and each entry is divided by *m*, producing the final average consensus matrix. Each entry in the final consensus matrix represents the fraction of times that two cells belong to the same cluster, ranging from 0 to 1. The *i*-th row of the final consensus matrix can be interpreted as the similarity between the *i*-th cell versus all other cells.
4. Similar cells (neighbors) for imputation are defined based on the final consensus matrix. For each cell *i*, the similarities with other cells are given by the entries in the *i*-th row of the final consensus matrix, denoted as ${\tilde{w}}_{i}$. A cutoff *c* is applied to define neighbors. The final weight $w_{i}$ is defined as
  (2) $$w_{ij}=\left\{\begin{array}{cc} {\tilde{w}}_{ik}, & \mathrm{if}\;{\tilde{w}}_{ik}\geq c,\;\mathrm{meaning}\;\mathrm{cell}\;k\;\mathrm{is}\;\mathrm{the}\;\mathrm{neighbor}\;\mathrm{of}\;\mathrm{cell}\;i;\\ 0, & \mathrm{otherwise}. \end{array}\right.$$
5. Zero counts are imputed using the final consensus matrix based on weighted or unweighted average. For the expression of the *i*-th cell and the *j*-th gene, the *i*-th row of the consensus matrix is extracted as weight $w_{\mathrm{ik}}$, where *k* represents a neighboring cell. The weighted estimate of expression ${\hat{x}}_{ij}$ for the *i*-th cell and the *j*-th gene is
  (3) $${\hat{x}}_{ij}=\frac{\sum_{k\neq i}w_{ik}x_{kj}}{\sum_{k\neq 0}w_{ik}+\epsilon },$$
  where ϵ is a small constant to avoid the zero denominator, $x_{kj}$ is the expression for the *k*-th cell and the *j*-th gene, and $w_{ik}$ is the (i,k)-th entry in the consensus matrix.
  The unweighted estimate of expression ${\hat{x}}_{ij}$ for the *i*-th cell and the *j*-th gene is
  (4) $${\hat{x}}_{ij}=\frac{\sum_{k\neq i}I\left(w_{ik}\neq 0\right)x_{kj}}{\sum_{k\neq 0}I\left(w_{ik}\neq 0\right)+\epsilon }.$$

More explanations of CCI algorithms are described Section 2.1. Since CCI provides flexibility with several parameters and options, we tune parameters and choose the optimal options for CCI algorithm, as described later in Section 5.2.

### 5.2. Sensitivity Analysis for Tuning Parameters

CCI offers several tunable parameters to optimize its performance. In Step 1, the proportion of sampled genes *p* should be specified. Lower proportions introduce more variability in clustering outcomes across iterations. In addition, sampling can be performed either from all genes or from the top variable genes. Including more genes may capture diverse biological features but also introduce noise and increase computational burden. In contrast, focusing on top variable genes reduces noise but risks missing subtle signals, making it essential to balance feature coverage and efficiency.

In Step 2, K-means clustering is applied to each subset sampling from the original data due to computational efficiency. In K-means, the number of groups *k* must be specified. A higher *k* focuses on capturing local structures, while a lower *k* emphasizes global clustering patterns.

In Step 3, the number of subsets *m* must be specified. A higher number of subsets enhances the accuracy and avoids the randomness of the consensus matrix, but also increases computational cost.

In Step 4, CCI defines cellular neighbors through the consensus matrix, where a cutoff *c* controls the inclusion of neighbors. Higher cutoffs limit the number of neighbors contributing to imputation, emphasizing local data structures. Lower cutoffs allow more neighbors, capturing broader patterns.

Sensitivity analysis evaluates how changes in input parameters impact the performance of a model, helping to identify which parameters significantly influence results and which have minimal impact. This process ensures that the model is well tuned, robust, and delivers consistent and reliable performance across different settings.

For sensitivity analysis, we conducted simulation studies for the following parameters:

- Proportion of sampling p={0.2,0.4,0.6,0.8};
- Number of genes sampled ngenes={100,200,300,all};
- Number of groups k={2,3,4,5,6};
- Cutoff for neighbors c={0,0.2,0.4,0.6,0.8};
- Number of samples m={10,50,100,200}.

**Figure 8:**
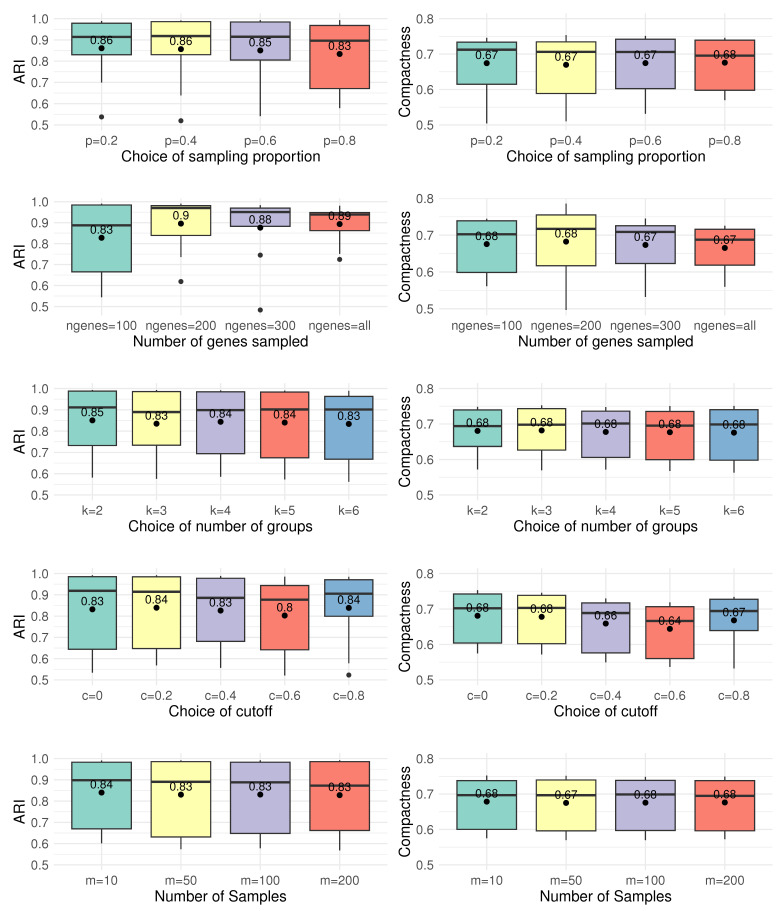
Impact of tuning parameters on clustering performance. ARI and compactness are used to assess the robustness of CCI. Panels display the effects of varying the sampling proportion *p*, the number of genes sampled ngenes, the number of groups *k*, the cutoff *c*, and the number of samples *m* on clustering outcomes. CCI demonstrates stable performance across a range of parameter values, indicating effective trade-offs between computational efficiency and clustering accuracy.

We mainly focus on the clustering performance, as it directly impacts downstream analyses like DE analysis. ARI and compactness metrics are used to assess clustering quality, as shown in Figure 8.

The results demonstrate that CCI is robust to the choice of proportion of sampling *p*, the number of groups *k* for clustering, and the cutoff *c*, indicating stable performance across these parameters. In addition, CCI shows effectiveness even with a small number of iterations (m=10), which can be used to enhance the computational efficiency. However, CCI exhibits some variation with different numbers of sampled genes. Although selecting the top 100 genes does not yield the highest ARI, it achieves the highest compactness while significantly reducing computational cost. This makes it the optimal choice for balancing performance and efficiency in the analysis.

Based on these results, the following parameter settings are used for the simulation studies in Section 2:

- Proportion of sampling p=0.8 from the top 100 variable genes;
- Number of groups k=4, avoiding assumptions about the true number of clusters;
- Cutoff for neighbors c=0.2 to define neighboring cells;
- Number of samples m=50, balancing computational efficiency with reduced randomness.

### 5.3. Simulation Settings

Raw count scRNA-seq data are simulated using the Splat model from the R package *Splatter* [12]. The ‘splatSimulateGroups’ function is used for simulating two groups of data in Section 2. It allows us to specify DE parameters as well as dropout parameters. The DE signal levels are controlled by the ‘de.facLoc’ parameter, which generates the differential expression for base expression levels for selected genes. It controls how similar groups are to each other. Lower values for this parameter result in higher similarity between groups, corresponding to weaker DE signals.

The severity of dropouts is controlled using the ‘dropout.mid’ parameter, which controls the probability that a particular count in a particular cell is set to zero. It is related to the mean expression of that gene in that cell, and this relationship can be modeled using a logistic function with dropout parameters. Lower values for this parameter corresponds to weaker dropout levels.

To comprehensively evaluate CCI and competing methods, we simulate three levels for signals and three levels for dropouts. For DE signals, we set the parameter ‘de.facLoc’ to 0.3, 0.2, and 0.1 to simulate strong, moderate, and weak signals, respectively. For dropouts, we specify the parameter ‘dropout.mid’ to 5, 4, and 2 for strong, moderate, and weak dropouts, respectively. These three dropout parameters result in 76%, 62%, and 32% dropout rates.

Figure 5 demonstrates the clustering patterns observed in the simulated data across different dropout and signal levels, under both SCTransform and log normalization. Without dropout events, two groups of data are well separated. As the dropout becomes stronger or the DE signal becomes weaker, the clustering pattern becomes less distinct, with the ARI decreasing accordingly.

For each simulation, we generate 2000 cells per group and 500 genes per dataset. To ensure the robustness of the results, we generate 200 datasets for each simulation setting. For every downstream analysis, the key metrics are averaged over these 200 repetitions to assess the overall performance and stability of each method. Additional simulation parameters are provided in Table 4 for reference.

### 5.4. Implementation of Competing Methods

RESCUE [5] applies a bootstrap-based sampling method to estimate mean expression by extracting the distribution of average expression levels across cells. RESCUE is implemented by the ‘bootstrapImputation’ function in R package *rescue*. The input of RESCUE should be a log-normalized expression matrix. After imputation, we scale the imputed data but avoid renormalization, as the original normalization has already been applied. The default number of bootstrap samples for RESCUE is 100, while for fair comparison, CCI uses 50 samples. Other RESCUE parameters are kept at their default settings.

scImpute [6] estimates each gene’s dropout probability per cell by fitting a mixture model, then imputes missing values using information from similar cells. scImpute is implemented by the ‘scimpute’ function in R package *scImpute*. The number of groups ‘Kcluster’ in scImpute is set to two, based on the truth that two clusters exist in both the simulated and real data studies. In contrast, CCI sets the number of groups to four, avoiding any assumptions about the true number of clusters.

DCA [7] uses an autoencoder to denoise the data with zero-inflated negative binomial likelihood as loss function. DCA is implemented by the Python package *dca*. In accordance with the recommendations from the DCA paper, we specify the ZINB loss function. All other parameters are kept at their default values.

### 5.5. Evaluations of Downstream Analyses

Existing methods for imputing or denoising dropouts often lack the comprehensive evaluation of their effectiveness in downstream analyses. In response to these challenges and weaknesses, we propose a comprehensive evaluation focusing on three downstream analyses: clustering, DE analysis, and single-genes evaluation. While clustering performance is commonly evaluated, DE analysis and single-gene assessments are frequently overlooked in most imputation methods. Additionally, numerical measures to quantify imputation performance are often neglected.

To evaluate clustering performance, dimension reduction plots are often used for visualizing the clustering pattern, such as PCA, UMAP [14], and tSNE [13]. These plots can be conveniently generated using the ‘RunPCA’, ‘RunUMAP’, and ‘RunTSNE’ functions in the R package *Seurat*. Adjusted Rand index (ARI) is used to quantify the clustering accuracy by measuring the similarity between predicted and true clustering labels [17]. ARI is an adjusted version of the Rand index (RI), representing the proportion of agreements over the total pairs. ARI lies between −1 and 1, with ARI=1 indicating identical clustering outcomes and ARI=−1 denoting completely dissimilar clusters.

However, it is important to note that two clusterings may yield the same ARI but exhibit different clustering patterns. For instance, one clustering may show tight groups while another may have dispersed groups. To capture these differences, we introduce the concept of compactness, which measures the relative distance between identified groups. Compactness is defined as the ratio of between-group variance B to the total variance, where total variance is the sum of between-group variance B and within-group variance W. Compactness values range from 0 to 1, with higher values indicating that the groups are more separated, which is desirable for clear clustering patterns. Between-group variance and within-group variance [18], as well as compactness, are defined as

$$B=\sum_{k=1}^{K}n_{k}{∥{\bar{x}}_{k}-\bar{x}∥}_{2}^{2},$$

$$W=\sum_{k=1}^{K}\sum_{c(i)=k}{∥x_{i}-{\bar{x}}_{k}∥}_{2}^{2},$$

$$\mathrm{compactness}=\frac{B}{B+W}$$

where *k* denotes the group index, k=1,2,⋯,K; $n_{k}$ is the sample size for group *k*; $\bar{x}$ is the overall average of all points; and ${\bar{x}}_{k}$ is the average of points in group *k*.

For DE analysis, several statistical tests and methods are commonly used to identify DE genes, including *t*-test, Wilcoxon rank sum test, LIMMA, and DESeq2. Most of these tests can be implemented using the ‘FindMarkers’ function in R package *Seurat*, with the Wilcoxon rank sum test used as default. To quantify the similarity between the true DE genes and the declared DE genes after imputation, we calculate the Jaccard index [19] to measure how similar the two sets are, with higher values indicating greater overlap between the declared and true DE genes.

Sensitivity and specificity are also computed to evaluate DE analysis. Sensitivity is defined as the proportion of true DE genes correctly identified, reflecting the method’s ability to detect DE genes. Specificity measures the proportion of correctly identified non-DE genes, indicating the method’s capacity to avoid false positives.

Assume that *A* and *B* denote the declared DE gene sets and true DE gene sets, while Ω denotes the set of all genes. The Jaccard index, sensitivity, and specificity are

$$J\left(A,B\right)=\frac{\left|A⋂B\right|}{\left|A⋃B\right|}$$

$$\mathrm{sensitivity}=\frac{\left|A⋂B\right|}{\left|B\right|}$$

$$\mathrm{specificity}=\frac{\left|(\Omega \setminus A)⋂(\Omega \setminus B)\right|}{\left|(\Omega \setminus B)\right|}$$

To assess imputation performance at the single-gene level, we generate scatter plots comparing the ground truth values on the x-axis with the imputed values on the y-axis. A pattern closely aligned with the 45-degree line in these plots indicates a strong imputation performance, as it suggests that the imputed data closely resemble the ground truth. In addition to visual assessment, we perform correlation analysis to quantify the similarity between the imputed data and the ground truth. Specifically, we compute Spearman’s correlations for each gene and average these correlations across all true DE genes, providing a robust measure of overall similarity between the original and imputed data.

### 5.6. Examination of Normalization Methods

In scRNA-seq data, systematic technical variability often arises due to differences in sequencing depth or inconsistencies in library preparation across cells. Normalization is essential to correct these technical biases, ensuring fair comparisons of expression profiles across cells. This study utilizes two normalization methods provided in the R package *Seurat*: log normalization and SCTransform [20].

Log normalization is implemented through the ‘NormalizeData’ and ‘ScaleData’ functions in *Seurat*. The ‘NormalizeData’ function adjusts for differences in library size by dividing the feature counts of each cell by the total counts within that cell and multiplying by a scale factor (default = 10,000). This step ensures that gene expression values are not dominated by sequencing depth variations. A natural-log transformation (log1p) is then applied to stabilize variance and make the data more normally distributed. After normalization, the ‘ScaleData’ function scales and centers the expression values across cells by subtracting the mean expression and standardizing each feature. This additional scaling step ensures that all genes have comparable magnitudes, enhancing the performance of downstream analyses.

SCTransform is an alternative to the NormalizeData, FindVariableFeatures, ScaleData workflow in *Seurat*. It integrates normalization and variance stabilization into a single framework using a regularized negative binomial model. This model captures the relationship between gene expression and variance, removing unwanted sources of technical noise while preserving biologically meaningful variation.

To examine the impact of normalization, we first calculate Pearson correlations and Spearman’s correlations between the original data and the transformed data using log normalization and SCTransform. Both methods exhibit strong correlations (above 0.8), indicating that they preserve the underlying data structure well. However, these two normalization methods lead to noticeably different data patterns. As shown in Figure 5, clustering under SCTransform produces clearer separation compared to log normalization, leading to a significantly higher ARI. Specifically, under moderate dropouts, data after SCTransform still show a clear clustering pattern, whereas log normalization results in mixed groupings of cells.

Given that normalization methods can alter data patterns and influence downstream analyses, it is crucial to evaluate imputation methods under different normalization strategies. While our initial simulation studies in Section 2 use log normalization, we also assess the performance of CCI and other competing methods under SCTransform in Section 2.6. This dual evaluation allows us to test whether CCI can maintain its effectiveness and adaptability regardless of the chosen normalization method, ensuring robust performance across different data preprocessing strategies.

### 5.7. Details of Type-1 Diabetes Mouse Dataset

In the real data studies in Section 2, we use a type-1 diabetes mouse dataset. This dataset is used to determine the effect of CD137 (Tnfrsf9) deficiency on regulatory T and CD8 T cells, containing 27,998 genes and 3199 cells. The data are composed of two mice strains, NOD.Foxp3-EGFP (wild-type) and NOD.Tnfrsf9-/-.Foxp3-EGFP (knock-out). Cell-type annotation is performed using the *Seurat* pipeline based on canonical markers, and two primary populations were identified: CD4 and CD8 T cells.

Quality control is performed on the type-1 diabetes mouse dataset. Since the expression of CD4 and CD8 on mature T cells is generally considered to be mutually exclusive, we remove the CD4 cells with CD8 expression, as well as the CD8 cells with CD4 expression. In total, 183 cells are filtered in this way.

Normalization for this dataset is performed using SCTransform. Although in Section 2 we primarily focus on log normalization, SCTransform is the preferred method for real data. This is because SCTransform accounts for key technical biases, including sequencing depth, overdispersion, and batch effects. Real scRNA-seq data are inherently more prone to technical variability, and SCTransform effectively reduces these biases, ensuring that biological signals are preserved [20].

Since we do not know whether the zero counts are real dropout events or not, we introduce three levels of dropouts to the original data R package *Splatter*: no additional dropouts (apply methods directly to the original data), additional 3.8% dropouts by setting ‘dropout.mid’ to 1, and additional 4.9% dropouts by setting ‘dropout.mid’ to 2. The dimension reduction plots of original data with and without extra dropouts are shown in Figure 6.

### 5.8. Clustering Methods: Shared Nearest-Neighbor and K-Means

In Step 2 of CCI algorithm, a clustering method must be specified for constructing the consensus matrix. Two clustering options are provided: shared nearest-neighbor (SNN) and K-means.

SNN evaluates the similarity between cells by the number of neighbors in common, and assigns objects to a cluster that shares a large number of nearest neighbors [15]. SNN can be implemented using the ‘FindNeighbors’ and ‘FindClusters’ functions in R package *Seurat*.

K-means partitions *n* observations into *K* distinct, nonoverlapping clusters. It minimizes the variance within each cluster, ensuring that each observation is assigned to the cluster with the nearest mean. In the CCI algorithm, after sampling, we first run PCA and UMAP on each subset of the original expression matrix, and then apply K-means clustering to the resulting UMAP components. The two-dimensional UMAP components avoid K-means to calculate distances in the original high-dimensional space and, thus, speed up the clustering. K-means can be implemented using the kmeans function in R package *stats*.

In Section 2, we utilize K-means for clustering the subsets within the CCI algorithm due to its faster computational speed and improved clustering accuracy for the sampled subsets. After imputation, we use the SNN to evaluate clustering performance, ensuring consistency and comparability with the evaluation methods reported in papers of competing approaches.

## Figures and Tables

**Table 1:** Summary table of existing methods for dropouts to show whether this method evaluates the downstream analyses in the paper, including clustering, DE analysis, and single gene evaluation. The methods in Category 1 borrow and aggregate the information of neighbors, and those in Category 2 denoise the entire dataset based on parametric models.

Method	Category	Clustering	DE Analysis	Single-Gene
RESCUE	1	Covered	Covered	Partially covered
scImpute	1	Covered	Covered	Partially covered
DrImpute	1	Partially covered	Not covered	Not covered
DCA	2	Not covered	Covered	Partially covered
SAVER	2	Covered	Partially covered	Covered
MAGIC	2	Covered	Not covered	Partially covered

**Table 2:** Summary of the averaged performance metrics for downstream analyses under nine simulation settings using the log normalization method. For each setting, 200 datasets are generated and the following metrics are computed: ARI, compactness, Jaccard index, and average Spearman’s correlation across true DE genes. Values are presented as the average of the means, and values in the parenthesis are the average of the standard deviations from nine settings (each consists of 200 repeats).

Method	ARI	Compactness	Jaccard Index	Spearman’s Corr
Without dropout	1 (0)	0.847 (0)	0.718 (0.004)	1 (0)
With dropout	0.57 (0.096)	0.502 (0.013)	0.459 (0.045)	0.400 (0.005)
KNN	0.73 (0.181)	0.544 (0.059)	0.322 (0.017)	0.404 (0.01)
DCA	0.515 (0.176)	0.387 (0.025)	0.277 (0.032)	0.241 (0.01)
RESCUE	0.868 (0.148)	0.698 (0.033)	0.497 (0.025)	0.417 (0.006)
scImpute	0.459 (0.2)	0.504 (0.032)	0.355 (0.059)	0.38 (0.009)
CCI	0.878 (0.082)	0.707 (0.025)	0.51 (0.014)	0.486 (0.002)

**Table 3:** Summary of the averaged performance metrics for downstream analyses under nine simulation settings using the SCTransform normalization method. For each setting, 200 datasets are generated and the following metrics are computed: ARI, compactness, Jaccard index, and average Spearman’s correlation across true DE genes. Values are presented as the average of the means, and values in the parenthesis are the average of the standard deviations from nine settings (each consists of 200 repeats).

Method	ARI	Compactness	Jaccard Index	Spearman’s Corr
Without dropout	1 (0)	0.847 (0.002)	0.37 (0.018)	1 (0)
With dropout	0.830 (0.157)	0.751 (0.028)	0.268 (0.042)	0.416 (0.004)
KNN	0.01 (0.038)	0.127 (0.027)	0.139 (0.035)	0.437 (0.005)
DCA	0.667 (0.168)	0.544 (0.03)	0.206 (0.01)	0.328 (0.01)
RESCUE	0.676 (0.155)	0.567 (0.022)	0.258 (0.058)	0.422 (0.007)
scImpute	0.656 (0.086)	0.577 (0.048)	0.228 (0.055)	0.435 (0.004)
CCI	0.891 (0.162)	0.681 (0.039)	0.285 (0.044)	0.509 (0.003)

**Table 4:** Simulation parameters for generating scRNA-seq data using R package *Splatter*.

Parameter	Intepretation	Value
seed	Random seed	Index of iteration
ngroups	Number of groups	2
batchCells	Cells per batch	2000
nGenes	Number of genes	500
de.prob	DE probability	0.1
		0.1 (weak signal)
de.facLoc	DE factor location	0.2 (moderate signal)
		0.3 (strong signal)
de.facScale	DE factor scale	0.4
de.downProb	Down-regulation probability	0.5
dropout.type	Dropout type	“experiment”
		2 (weak dropout)
dropout.mid	Dropout mid point	4 (moderate dropout)
		5 (strong dropout)
dropout.shape	Dropout shape	−1

## Data Availability

CCI is implemented in R and is available at https://github.com/wanlinjuan/CCI (accessed on 29 December 2024).

## Supplementary Materials

The following supporting information can be downloaded at: https://www.mdpi.com/article/10.3390/bioengineering12010031/s1, Figure S1: Box plots of ARI under log normalization method using data without dropouts, data with dropouts, and imputed data using CCI or competing methods. Figure S2: Box plots of compactness under log normalization method using data without dropouts, data with dropouts, and imputed data using CCI or competing methods. Figure S3: Box plots of Jaccard index under log normalization method using data without dropouts, data with dropouts, and imputed data using CCI or competing methods. Figure S4: Box plots of Spearman’s correlation across true DE genes under log normalization method using data without dropouts, data with dropouts, and imputed data using CCI or competing methods. Figure S5: Box plots of ARI under SCTransform normalization method using data without dropouts, data with dropouts, and imputed data using CCI or competing methods. Figure S6: Box plots of compactness under SCTransform normalization method using data without dropouts, data with dropouts, and imputed data using CCI or competing methods. Figure S7: Box plots of Jaccard index under SCTransform normalization method using data without dropouts, data with dropouts, and imputed data using CCI or competing methods. Figure S8: Box plots of Spearman’s correlation across true DE genes under SCTransform normalization method using data without dropouts, data with dropouts, and imputed data using CCI or competing methods.

## References

[1] Potter S.S. (2018). Single-cell RNA sequencing for the study of development, physiology and disease. Nat. Rev. Nephrol..

[2] Gong W., Kwak I.Y., Pota P., Koyano-Nakagawa N., Garry D.J. (2018). DrImpute: Imputing dropout events in single cell RNA sequencing data. BMC Bioinform..

[3] Hicks S.C., Townes F.W., Teng M., Irizarry R.A. (2018). Missing data and technical variability in single-cell RNA-sequencing experiments. Biostatistics.

[4] Zhang L., Zhang S. (2018). Comparison of computational methods for imputing single-cell RNA-sequencing data. IEEE/ACM Trans. Comput. Biol. Bioinform..

[5] Tracy S., Yuan G.C., Dries R. (2019). RESCUE: Imputing dropout events in single-cell RNA-sequencing data. BMC Bioinform..

[6] Li W.V., Li J.J. (2018). An accurate and robust imputation method scImpute for single-cell RNA-seq data. Nat. Commun..

[7] Eraslan G., Simon L.M., Mircea M., Mueller N.S., Theis F.J. (2019). Single-cell RNA-seq denoising using a deep count autoencoder. Nat. Commun..

[8] Van Dijk D., Sharma R., Nainys J., Yim K., Kathail P., Carr A.J., Burdziak C., Moon K.R., Chaffer C.L., Pattabiraman D. (2018). Recovering gene interactions from single-cell data using data diffusion. Cell.

[9] Lopez R., Regier J., Cole M.B., Jordan M.I., Yosef N. (2018). Deep generative modeling for single-cell transcriptomics. Nat. Methods.

[10] Monti S., Tamayo P., Mesirov J., Golub T. (2003). Consensus clustering: A resampling-based method for class discovery and visualization of gene expression microarray data. Mach. Learn..

[11] Zhu X., Zhang J., Xu Y., Wang J., Peng X., Li H.D. (2020). Single-cell clustering based on shared nearest neighbor and graph partitioning. Interdiscip. Sci. Comput. Life Sci..

[12] Zappia L., Phipson B., Oshlack A. (2017). Splatter: Simulation of single-cell RNA sequencing data. Genome Biol..

[13] Van der Maaten L., Hinton G. (2008). Visualizing data using t-SNE. J. Mach. Learn. Res..

[14] McInnes L., Healy J., Melville J. (2018). Umap: Uniform manifold approximation and projection for dimension reduction. arXiv.

[15] Kumari S., Maurya S., Goyal P., Balasubramaniam S.S., Goyal N. (2016). Scalable parallel algorithms for shared nearest neighbor clustering. Proceedings of the 2016 IEEE 23rd International Conference on High Performance Computing (HiPC).

[16] Stegle O., Teichmann S.A., Marioni J.C. (2015). Computational and analytical challenges in single-cell transcriptomics. Nat. Rev. Genet..

[17] Rand W.M. (1971). Objective criteria for the evaluation of clustering methods. J. Am. Stat. Assoc..

[18] Hastie T. (2009). The elements of statistical learning: Data mining, inference, and prediction. J. R. Stat. Soc..

[19] Chung N.C., Miasojedow B., Startek M., Gambin A. (2019). Jaccard/Tanimoto similarity test and estimation methods for biological presence-absence data. BMC Bioinform..

[20] Hafemeister C., Satija R. (2019). Normalization and variance stabilization of single-cell RNA-seq data using regularized negative binomial regression. Genome Biol..

