# Supplementary material for: CCI: A Consensus Clustering-Based Imputation Method for Addressing Dropout Events in scRNA-Seq Data
Source: Bioengineering (Basel). 2025 Jan 3;12(1):31. doi: 10.3390/bioengineering12010031 (PMC11763284; doi:10.3390/bioengineering12010031)
Supplement: Supplementary file 1 [file bioengineering-12-00031-s001.zip › bioengineering-3357914-supplementary.pdf]

CCI: A Consensus Clustering-Based Imputation Method for Addressing Dropout Events in scRNA-seq Data

December 15, 2024

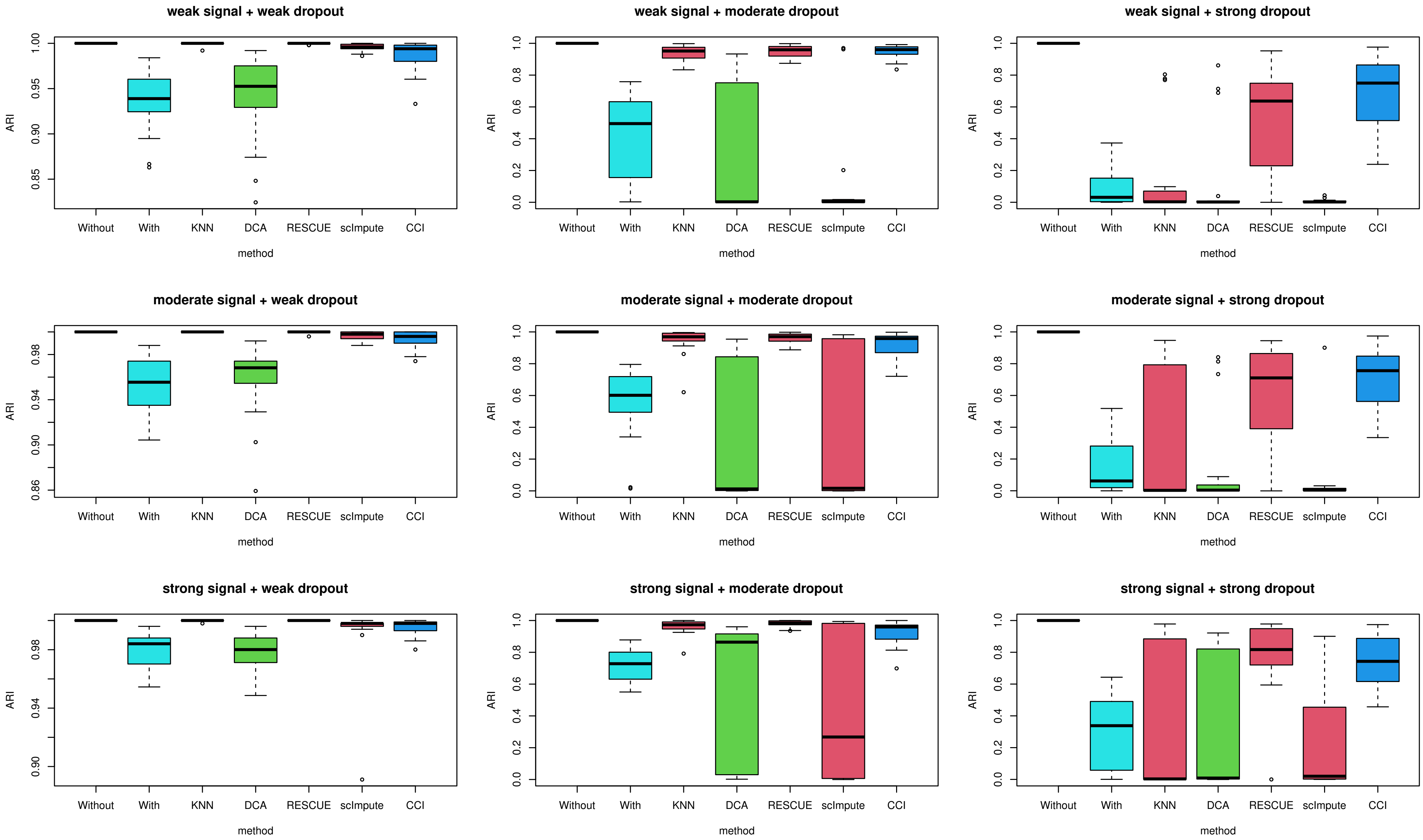

Supplementary Figure S1: Boxplots of ARI under log normalization method using data without dropouts, data with dropouts and imputed data using CCI or competing methods.

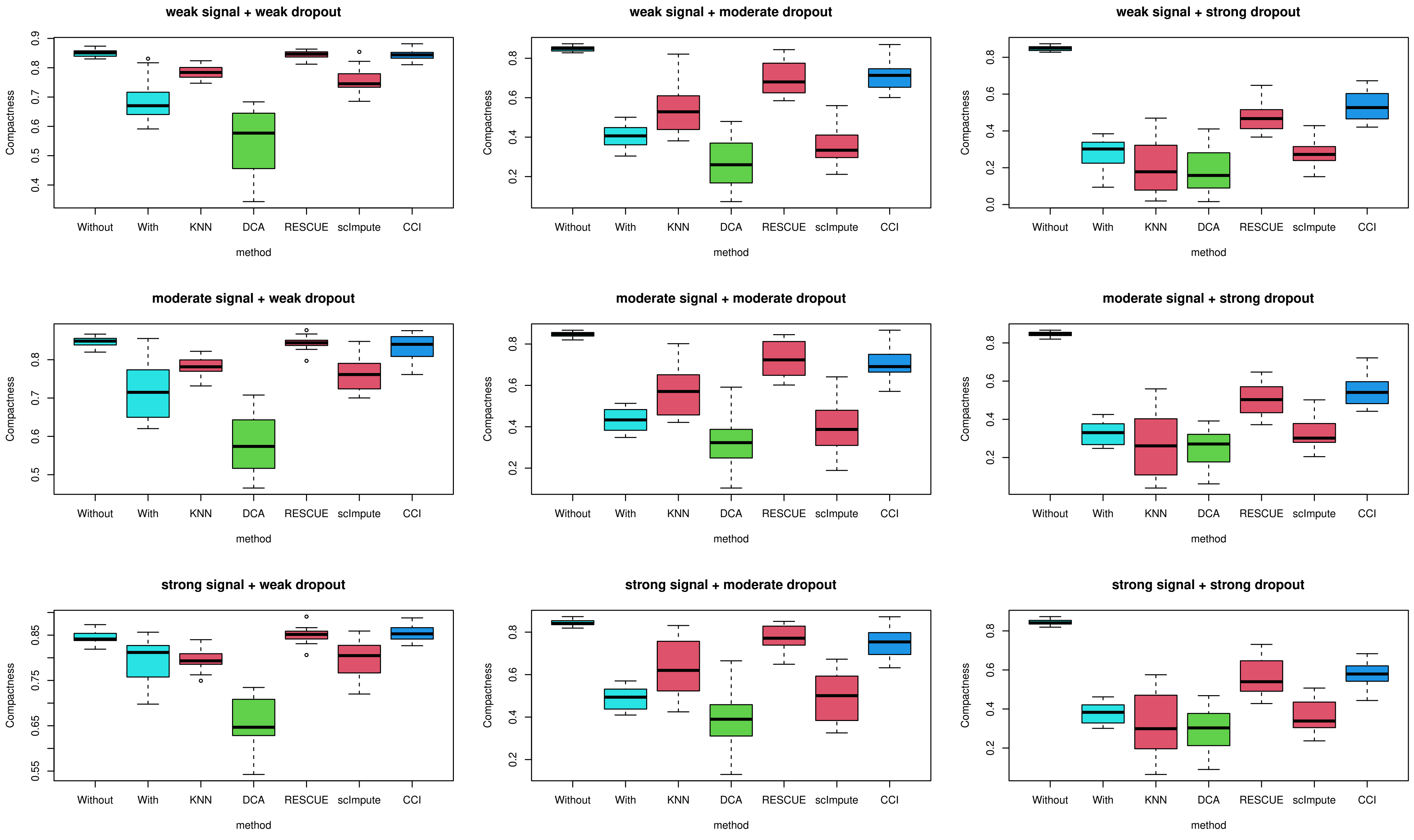

Supplementary Figure S2: Boxplots of compactness under log normalization method using data without dropouts, data with dropouts and imputed data using CCI or competing methods.

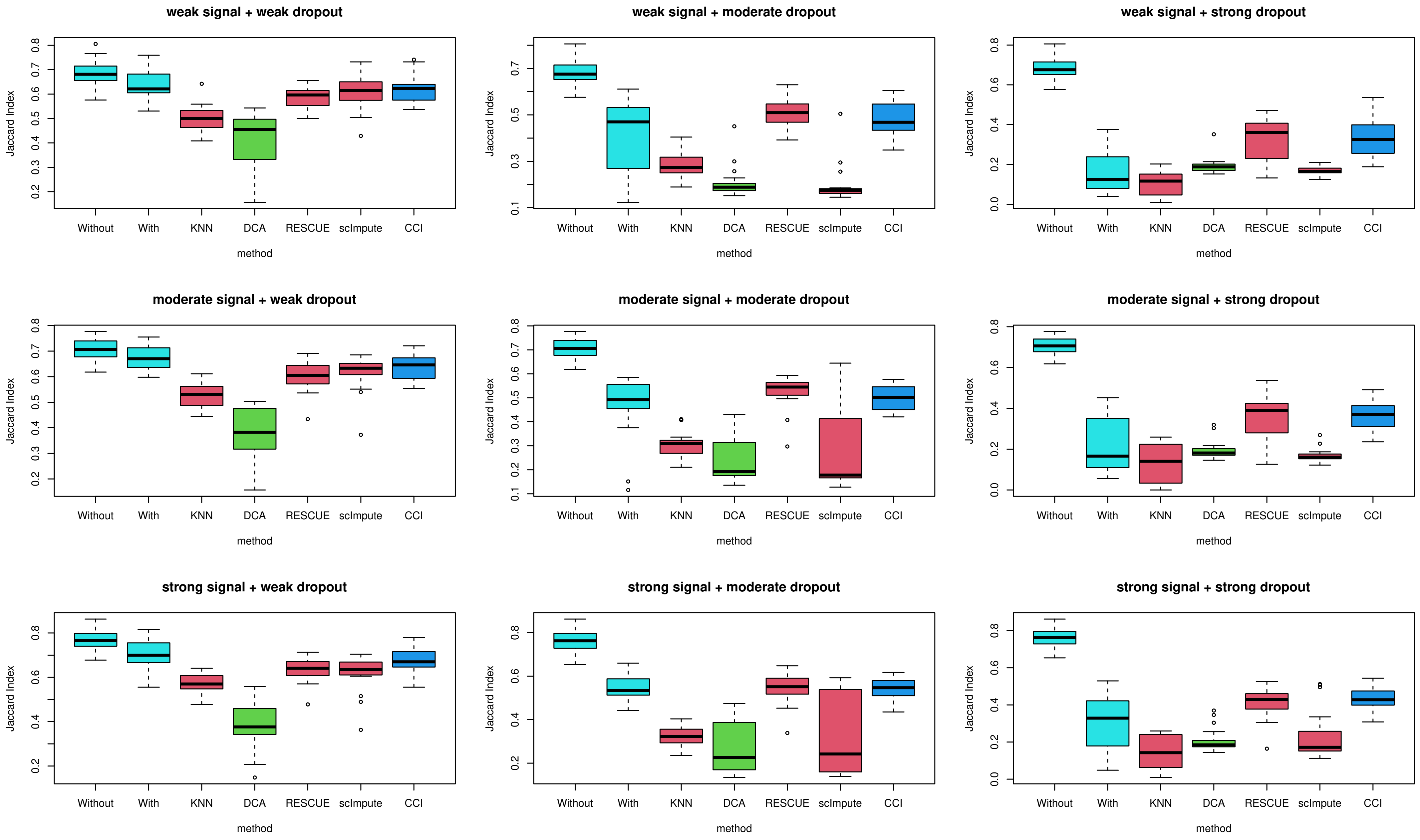

Supplementary Figure S3: Boxplots of Jaccard Index under log normalization method using data without dropouts, data with dropouts and imputed data using CCI or competing methods.

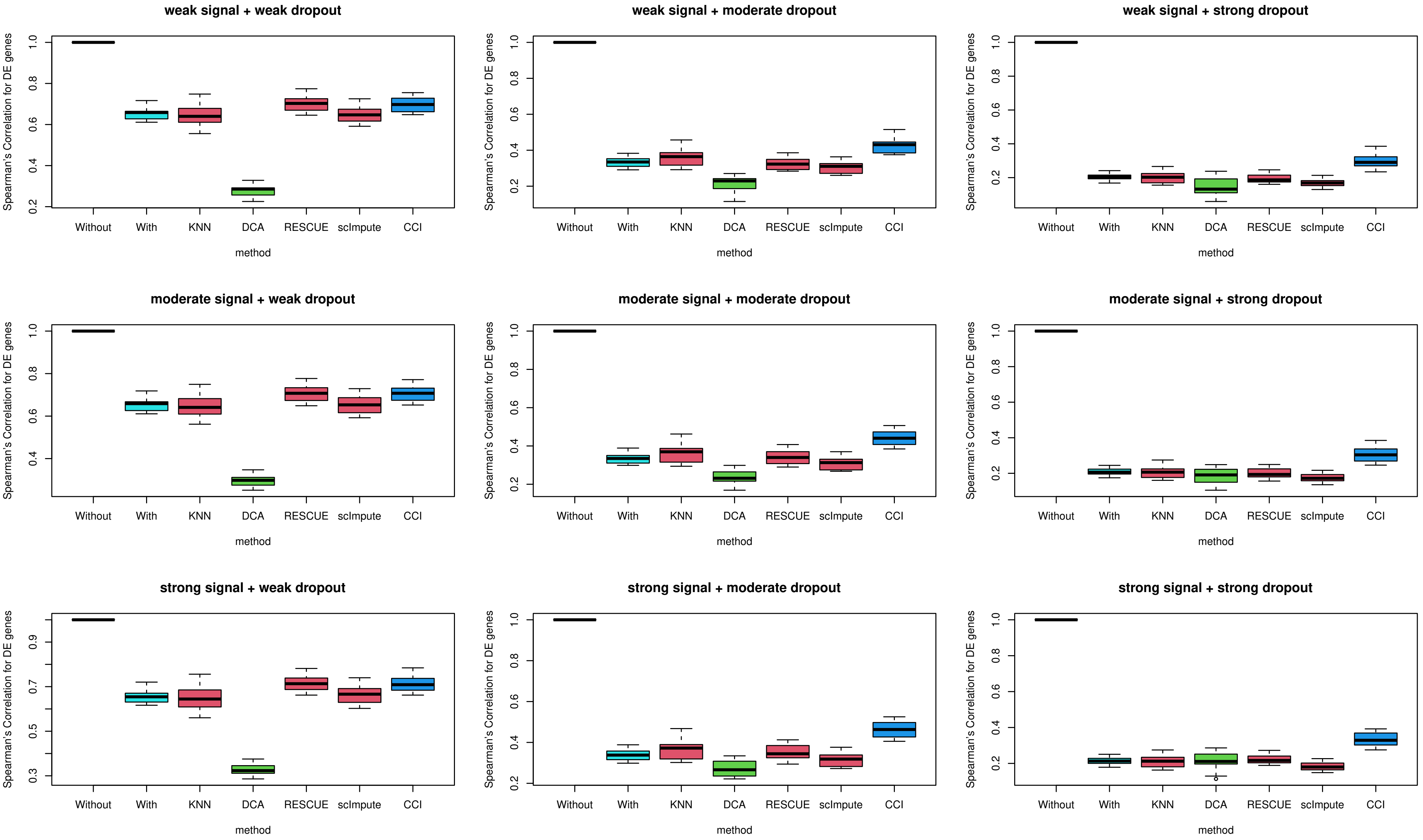

Supplementary Figure S4: Boxplots of Spearman's correlation across true DE genes under log normalization method using data without dropouts, data with dropouts and imputed data using CCI or competing methods.

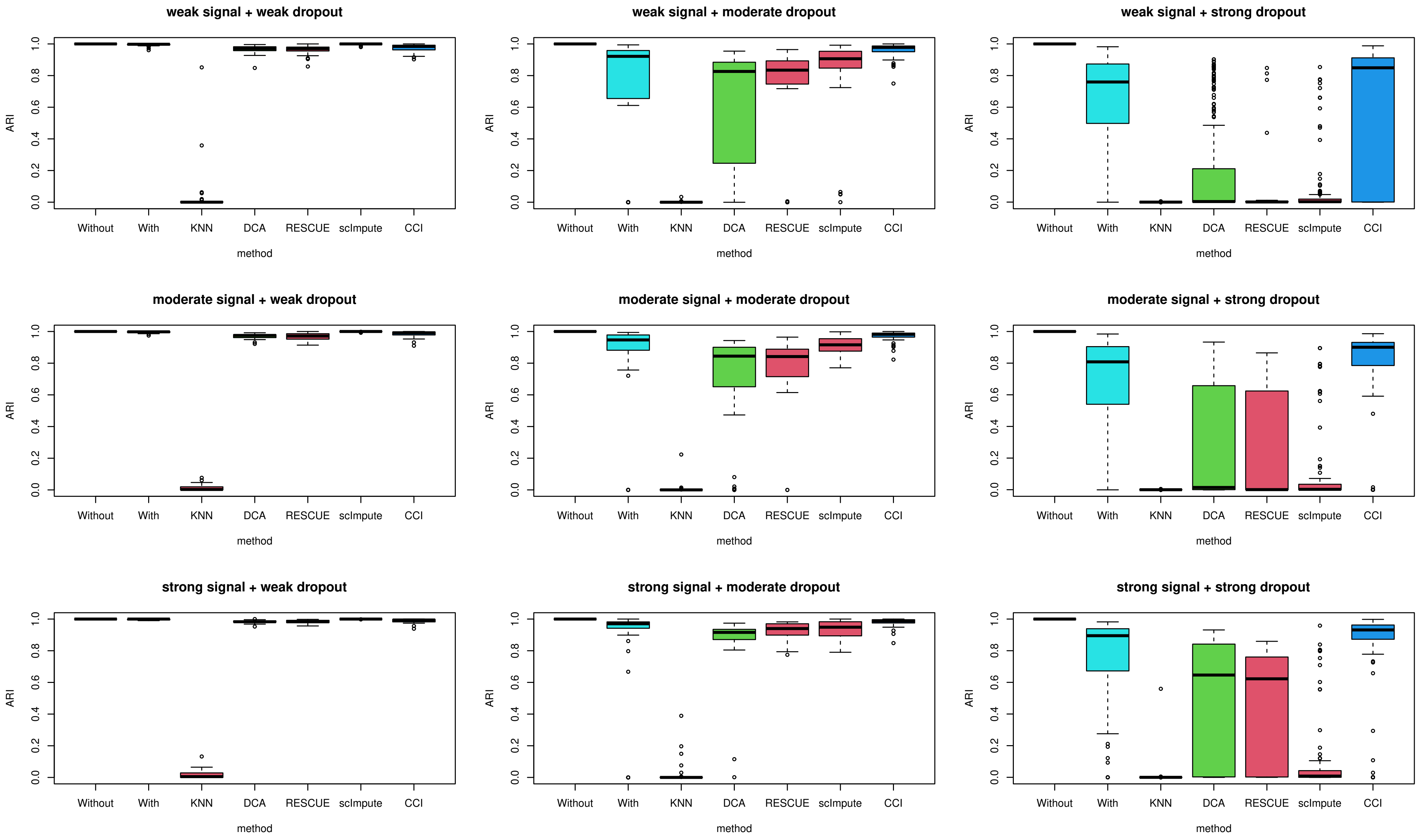

Supplementary Figure S5: Boxplots of ARI under SCTransform normalization method using data without dropouts, data with dropouts and imputed data using CCI or competing methods.

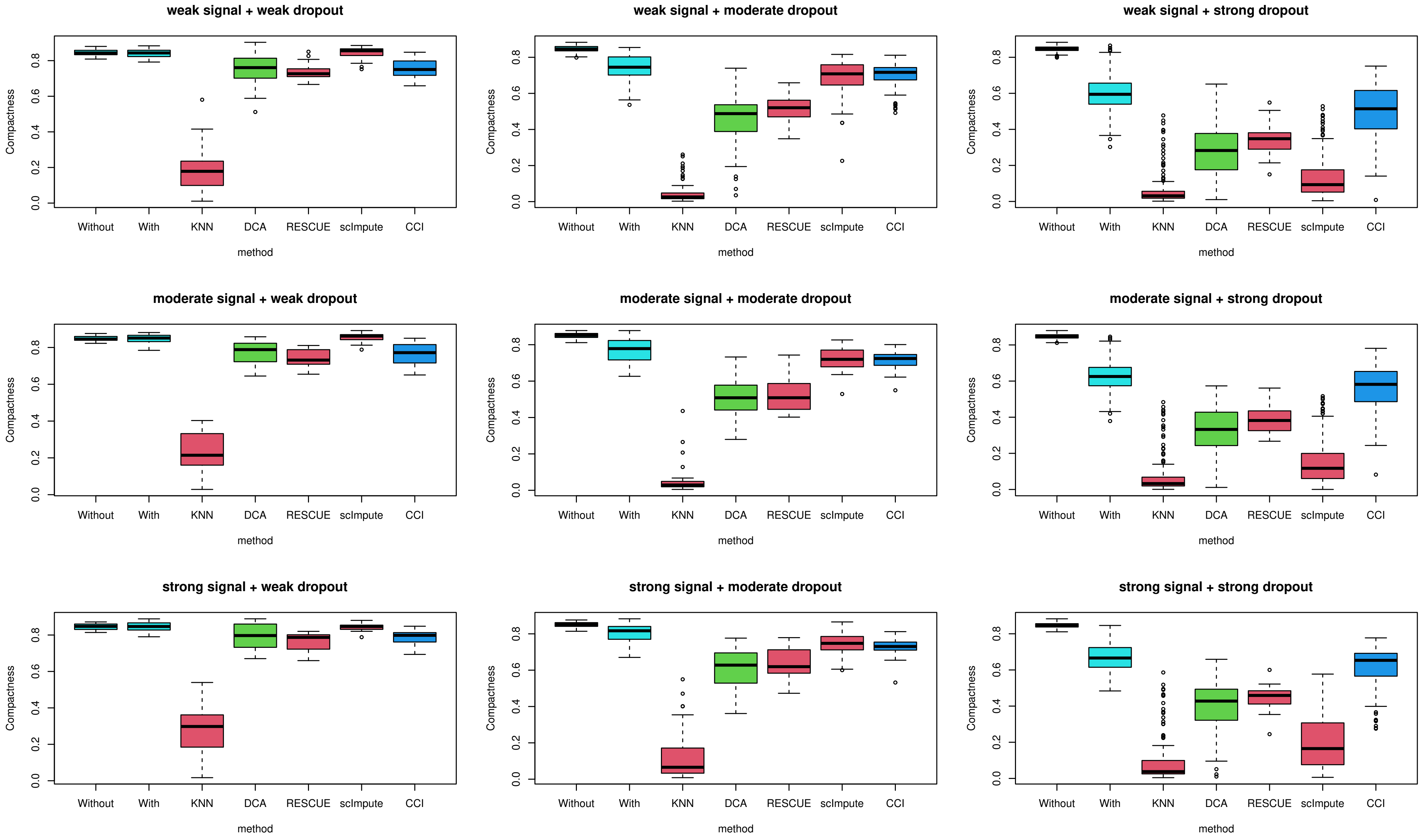

Supplementary Figure S6: Boxplots of compactness under SCTransform normalization method using data without dropouts, data with dropouts and imputed data using CCI or competing methods.

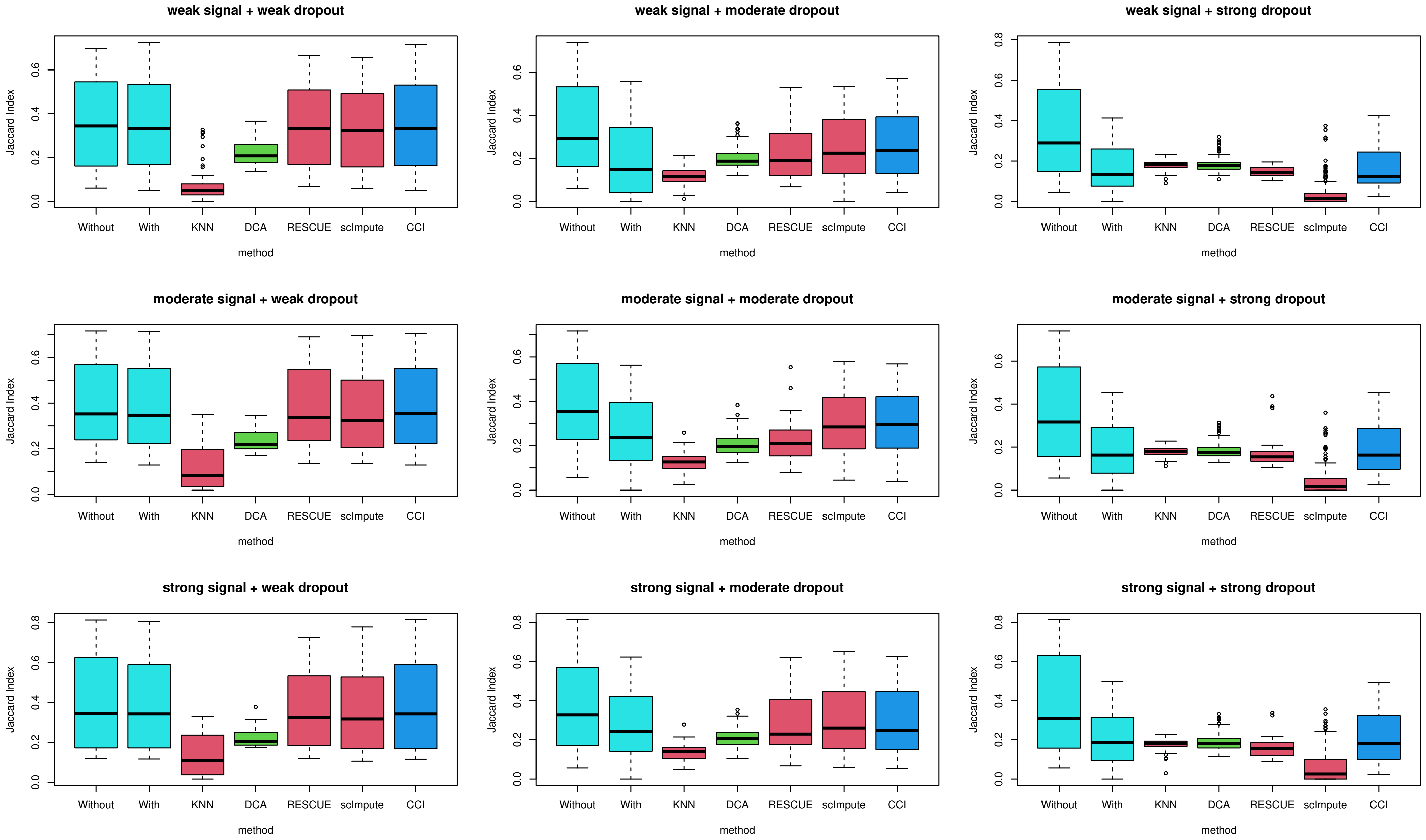

Supplementary Figure S7: Boxplots of Jaccard Index under SCTransform normalization method using data without dropouts, data with dropouts and imputed data using CCI or competing methods.

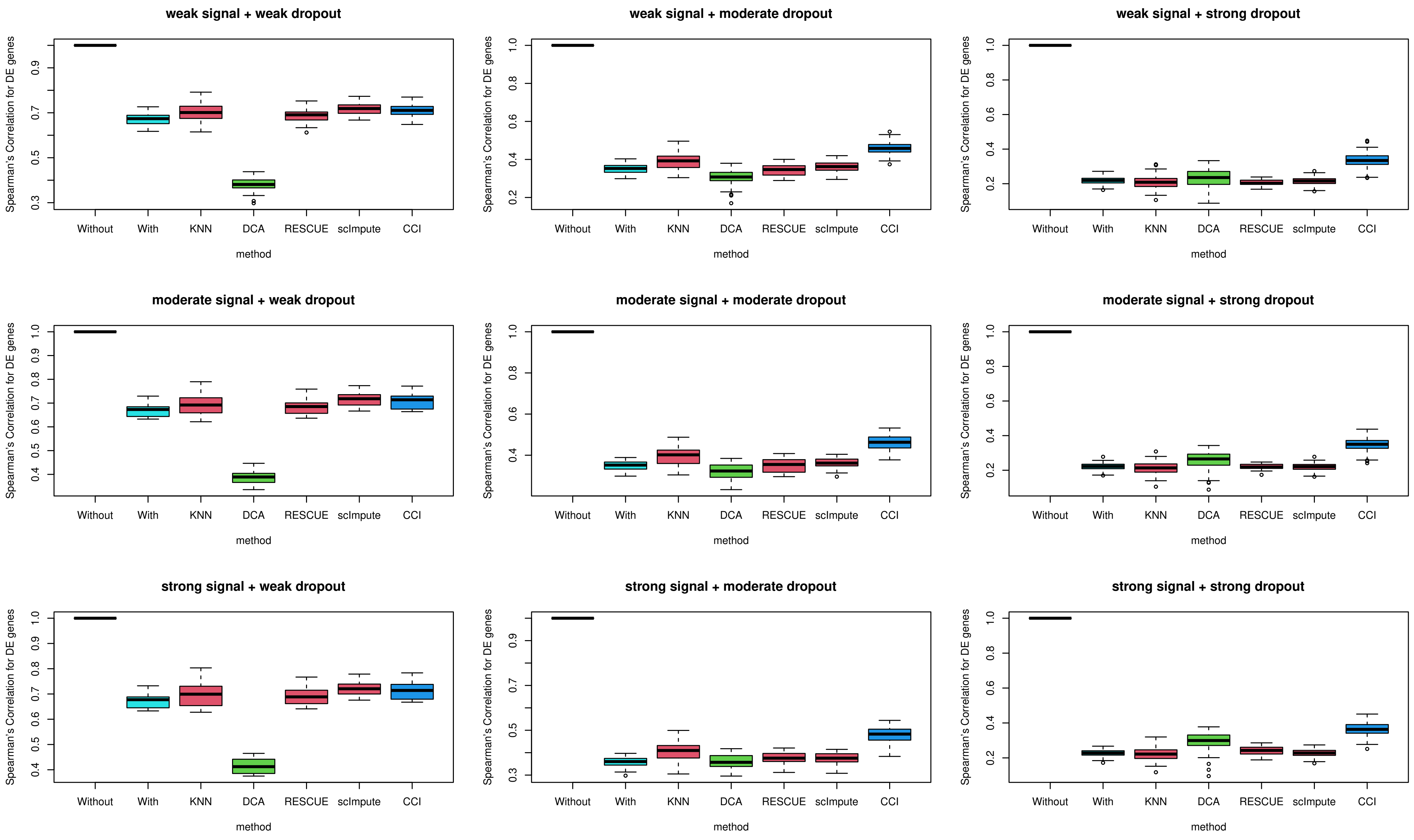

Supplementary Figure S8: Boxplots of Spearman's correlation across true DE genes under SCTransform normalization method using data without dropouts, data with dropouts and imputed data using CCI or competing methods.
